# The polar Ras-like GTPase MglA activates type IV pilus via SgmX to enable twitching motility in *Myxococcus xanthus*

**DOI:** 10.1073/pnas.2002783117

**Published:** 2020-10-22

**Authors:** Romain Mercier, Sarah Bautista, Maëlle Delannoy, Margaux Gibert, Annick Guiseppi, Julien Herrou, Emilia M. F. Mauriello, Tâm Mignot

**Affiliations:** ^a^Aix-Marseille Université-CNRS (UMR7283), Laboratoire de Chimie Bactérienne, Institut de Microbiologie de la Méditerranée, 13009 Marseille, France

**Keywords:** type IV pilus, cell motility, small GTPase, *Myxococcus xanthus*

## Abstract

Type IV pili (Tfp) are highly conserved macromolecular structures that fulfill diverse cellular functions, such as adhesion to host cells, the import of extracellular DNA, kin recognition, and cell motility (twitching). Outstandingly, twitching motility enables a poorly understood process by which highly coordinated groups of hundreds of cells move in cooperative manner, providing a basis for multicellular behaviors, such as biofilm formation. In the social bacteria *Myxococcus xanthus*, we know that twitching motility is under the dependence of the small GTPase MglA, but the underlying molecular mechanisms remain elusive. Here we show that MglA complexed to GTP recruits a newly characterized Tfp regulator, termed SgmX, to activate Tfp machines at the bacterial cell pole. This mechanism also ensures spatial regulation of Tfp, explaining how MglA switching provokes directional reversals. This discovery paves the way to elucidate how polar Tfp machines are regulated to coordinate multicellular movements, a conserved feature in twitching bacteria.

## Significance

The type IV pilus (Tfp) is a multipurpose machine found on bacterial surfaces that works by cycles of synthesis/retraction of a pilin fiber. During surface (twitching) motility, the coordinated actions of multiple Tfps at the cell pole promotes single cells and synchronized group movements. Here, directly observing polar Tfp machines in action during motility of *Myxococcus xanthus*, we identified the mechanism underlying pole-specific Tfps activation. In this process, the Ras-like protein MglA targets a novel essential Tfp-activator, SgmX, to the pole, ensuring both the unipolar activation of Tfps and its switching to the opposite pole when cells reverse their movement. Thus, a dynamic cascade of polar activators regulates multicellular movements, a feature that is likely conserved in other twitching bacteria.

To survive in their environment, interact with their host during infection, and create complex communities, bacteria have evolved a wide range of sophisticated surface nanomachines (e.g., flagella, secretion systems, surface pili). Among them, the type IV pilus (Tfp), a subclass of the type IV filament (Tff) superfamily, includes the type II secretion system (T2SS), the gram-positive competence pilus and archaellum in Archaea. Tfps are particularly widespread in bacteria and play key roles in adaptation, development, and virulence (1, 2). While Tfps exhibit highly conserved macromolecular structures, they support very diverse cellular functions, including host cell adhesion, extracellular DNA import (competence), kin recognition, and cell motility (twitching) (1–3). Twitching motility leads to the remarkable formation of coordinated cell groups, in which hundreds of cells move in a cooperative manner similar to swarming motility in flagellated bacteria (4, 5).

Recently, Tfps were directly observed by cryo-electron tomography in intact cells (6–8). Combined with a wealth of structural and genetic works, a global architecture of Tfp subtype a (Tfpa) has been proposed at molecular resolution. Tfpa commonly assemble into a multilayered structure that spans the entire cell envelope. Secretin (PilQ) forms the major outer membrane pore, required for pilin filaments to exit the cell envelope. The secretin is directly anchored to the peptidoglycan via its amidase N-terminal domain (AMIN) and the peptidoglycan-binding protein TsaP (9–11). The secretin is linked to the inner membrane (IM) parts via the periplasmic PilNOP complex, which extends coiled-coil domains into the IM to connect with the cytoplasmic ring protein PilM. The PilM ring might act as a chassis for a rotary shaft assembled by PilC. Depending on associated cytoplasmic motors (PilB and PilT), the rotation of PilC would then promote assembly (PilB-dependent) or deassembly (PilT-dependent) of the major pilin PilA subunits into filaments recruited at the periplasmic side of the Tfpa complex. This process results in extension and retraction cycles of µm-long pilin filaments, which can attach to a large variety of surfaces and substrates (1, 2).

In gram-negative bacteria, it is well understood that Tfpa acts as grappling hooks extending form the leading cell pole and pulling the cell as they retract (12, 13). The underlying molecular mechanisms are complex because multiple Tfpa machineries are present at the cell pole and must then be coordinated to promote persistent movements (12, 13). In addition, Tfpa machines must also be active at only one cell pole. How this is resolved is unclear, because Tfpa machines are presumably assembled during cell division by the action of the conserved peptidoglycan-binding AMIN domain of PilQ (9, 11), and, consequently, both poles contain preassembled Tfpa machines (11, 14–16). Therefore, persistent and directional movements require asymmetric activation and coordination at one cell pole. In some bacteria (e.g., *Myxococcus xanthus*; see below), regulation promotes the switch of polarity activation, thus allowing cells to rapidly change their direction (reversal) in response to signaling (17, 18). In the present work, we uncover the mechanism that mediates this pole-specific activation of Tfpa machines in *M. xanthus*.

*M. xanthus* motility plays a crucial role to swarm, predate on prey bacteria, and build differentiated multicellular structures (fruiting bodies) (19). Remarkably, this bacterium uses two different motility engines. The so-called gliding (A, adventurous) motility system promotes the motility of single cells at colony borders. At the molecular level, A-motility is driven by the recently identified Agl-Glt complex, which propels the cell as it moves along the cell axis and adheres at so-called bacterial focal adhesions (bFAs) (20). The *Myxococcus* cells can also organize into large motile cell groups (social [S] motility), which, as mentioned above, is a form of twitching motility (21). S-motility also requires exopolysaccharide (EPS) synthesis (22), which enables Tfpa pilin surface attachment and cell–cell interactions (23). Remarkably, both the A- and S-motility systems are assembled at the cell pole (the leading pole), which is spatially regulated by the switch protein MglA, a Ras-like G protein.

MglA binds to the leading pole only in a GTP-bound state, its active form (24). This unipolar distribution results from the combined actions of 1) the RomRX complex, a composite guanosine exchange factor (GEF) that recruits MglA at the leading cell pole and loads it with GTP (24), and 2) MglB, a GTPase-activating protein (GAP) that localizes at the opposite cell pole (lagging), where it deactivates MglA by provoking GTP hydrolysis (25, 26). RomRX, MglA, and MglB together form a polarity axis that can be inverted by signaling to promote rapid changes in the direction of movement (reversals). The switch itself has been thoroughly investigated and is provoked by a bacterial chemosensory-like apparatus (Frz system) (27, 28). In the case of A-motility, it is partially understood that MglA-GTP recruits the transenvelope Agl-Glt complex to a cytoplasmic platform formed by the MreB actin cytoskeleton and the AglZ protein, thereby forming so-called bacterial focal adhesions (29, 30). However, how MglA-GTP interacts with Tfpa is currently unknown.

In this study, we first developed a cysteine-labeling pilin method to follow major pilin filament dynamics during twitching motility and study direct connections between MglA-GTP and Tfpa activity. While doing so, we discovered that MglA is not strictly required for Tfpa function, but mediates pole-specific Tfpa machine activation. In this process, MglA-GTP interacts directly with a newly identified protein, SgmX, that promotes functional Tfpa pilin assembly as it becomes localized to the cell pole. Ultimately, our results reveal that dynamic protein activators regulate Tfpa machines spatially, which likely occurs in other twitching bacteria as well.

## Results

### MglA Regulates S-Motility by Pole-Specific Activation of Tfpa Machines.

**Fig. 1.**
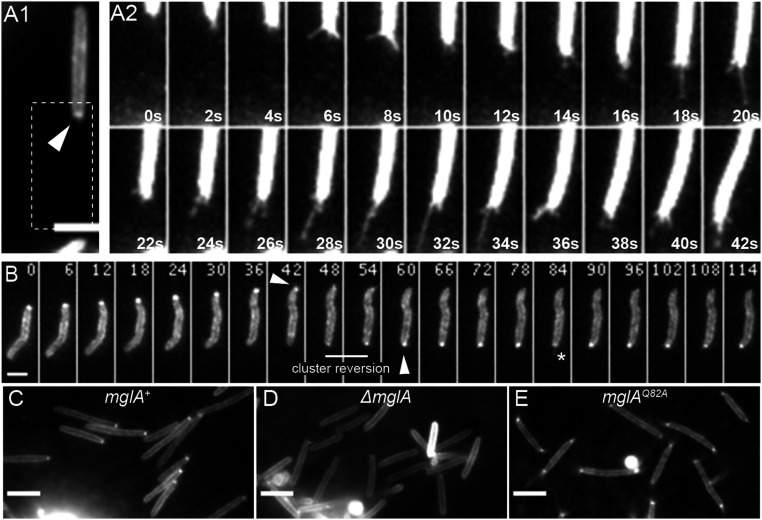
MglA-GTP regulates pole-specific Tfpa activation. (*A* and *B*) Dynamics of labeled Tfpa pilin filaments of the strain RM384 (DZ2 *att*^*mx8*^*::P*_*pilA*_*-pilA*^*D71C*^) observed by TIRF microscopy. *A2* represents enlarged time-lapse series of *A1* image (dashed rectangle). The arrow points to polar cluster enrichment of labeled pilin, and the star highlights the labeled pilin filament. Elapsed time (s) is shown in each panel. (Scale bars: 2 μm.) See also Movies S1–S3. (*C*–*E*) TIRF microscopy images of labeled Tfpa pilin of strains RM384 (*DZ2 att*^*mx8*^*::P*_*pilA*_*-pilA*^*D71C*^ (*C*), RM386 (*mglA*^*Q82A*^ *att*^*mx8*^*::P*_*pilA*_*-pilA*^*D71C*^ (*D*), and RM390 (*ΔmglA att*^*mx8*^*::P*_*pilA*_*-pilA*^*D71C*^ (*E*). (Scale bars: 4 μm.)

To investigate how MglA-GTP activates S-motility, we aimed to image Tfpa-pilin filament dynamics directly in single twitching cells. For that purpose, we adapted a recently described pilin cysteine-labeling method that involves adding extracellularly cysteine-reactive maleimide fluorescent conjugates; this technique enabled the real-time imaging of Tfps in other bacteria (3, 31, 32). Among several cysteine substitutions tested in the major pilin PilA subunit, we found one substitution, PilA^D71C^, that allowed us to label pilin filaments when expressed under the control of the *pilA* promoter (P_pilA_). Unfortunately, this variant *pilA*^*D71C*^ was poorly functional for motility (*SI Appendix*, Fig. S1*A*). To circumvent this problem, we expressed PilA^D71C^ in the presence of PilA^wt^ and test whether the maleimide-labeled pilin monomers could be incorporated into active pilin filaments. The resulting merodiploid strain was indeed motile on soft agar (*SI Appendix*, Fig. S1*A*) and dynamic, fluorescent pili could be visualized when cells where spotted on carboxymethylcellulose (CMC) (Fig. 1*A* and Movie S1), a surface that permits single cell twitching of *M. xanthus* cells (23, 33). Interestingly, two main features emerged from our observations: 1) labeled PilA^D71C^ pilin formed a specific fluorescence enrichment cluster at the active cell pole (Fig. 1 *A*–*C*, arrow), absent in a strain lacking the variant *pilA*^*D71C*^ (*SI Appendix*, Fig. S1*B*); 2) in motile cells, dynamics of the pili filaments could be directly observed at the leading cell pole, propelling the cell as they extended and retracted (Fig. 1*A2* and Movie S1) in a similar fashion as observed in *Pseudomonas aeruginosa* using other techniques (12, 13). Furthermore, during reversals, retractile pilin filaments disassembled from one pole and reassembled at the opposite cell pole, coinciding with the relocation of fluorescent pilin polar clusters (Fig. 1*B* and Movies S2 and S3). Along with direct observation of Tfpa-based motility reversal in a bacterium, our data identify a polar pilin pool as a hallmark of active Tfpa machines. We hypothesize that the asymmetric formation of the pilin polar pool could hint at the pole-specific activation of Tfpa machines.

Thanks to the fluorescent reporter system described above, we could then investigate the function of MglA in the formation of this polar pilin pool as well as the pilin filament dynamics. Remarkably, *mgla* cells do not form polar pilin clusters (Fig. 1*D*); however, real-time pili labeling revealed that *mglA* cells could still sporadically assemble pili filaments at both cell poles (Movie S4), suggesting that MglA per se is not strictly required for Tfpa function, but rather is implicated in pole-specific Tfpa machine activation. To confirm this hypothesis, we took advantage of an MglA^Q82A^ variant that cannot hydrolyze GTP and is symmetrically distributed at both poles (25, 26). Following the labeled pilins in this genetic background, we observed both polar pilin clusters and dynamic pilin filaments at both cell poles (Fig. 1*E* and Movie S5). We conclude that MglA-GTP triggers the activation of S-motility by promoting the recruitment of pilin subunits to form a polar pool, thus allowing the formation of dynamic filaments at the pole.

### Identification of MglA-Independent Motility Suppressor Variants.

**Fig. 2.**
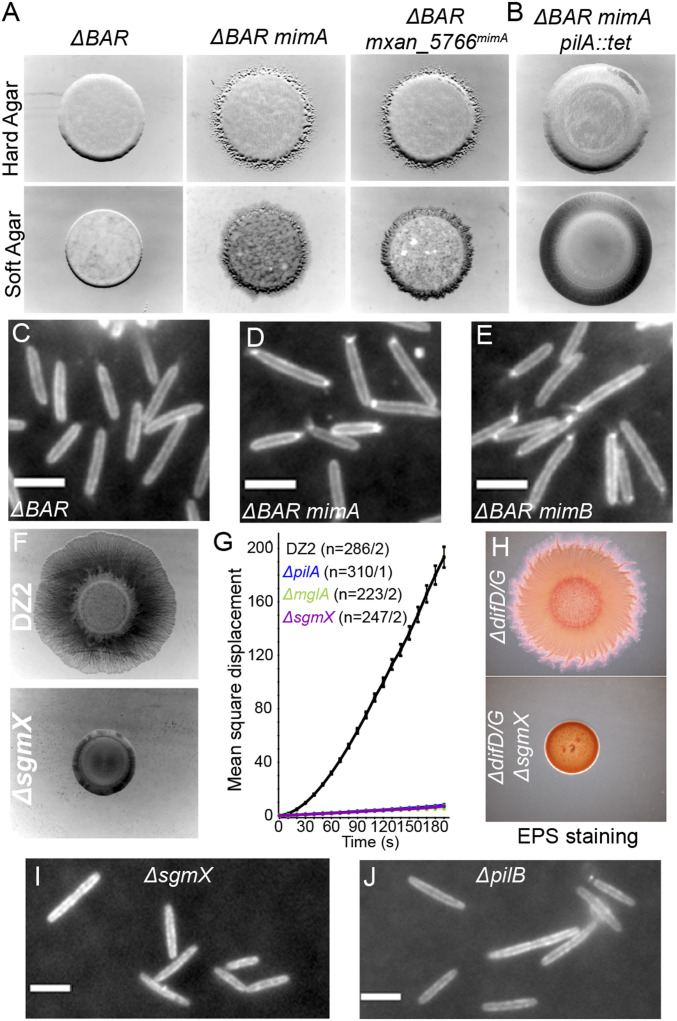
Mgla-independent motility variants unveil SgmX protein, an essential Tfpa activator. (*A*) Motility phenotypic assay of strains TM500 (*ΔBAR*; *Left*), RM55 (*ΔBAR* *mimA*; *Center*), and RM310 (*ΔBAR* *mxan_5766*^*mimA*^; *Right*) on hard (*Top*) and soft (*Bottom*) agar plates. (*B*) Motility phenotypic assay of the strain RM83 (*ΔBAR* *mimA pilA::tet*) on hard (*Top*) and soft (*Bottom*) agar plates. (*C*–*E*) TIRF microscopy images of labeled Tfpa pilin of strains RM388 (*ΔBAR att*^*mx8*^*::P*_*pilA*_*-pilA*^*D71C*^) (*C*), RM394 (*ΔBAR mimA att*^*mx8*^*::P*_*pilA*_*-pilA*^*D71C*^) (*D*), and RM395 (*ΔBAR mimB att*^*mx8*^*::P*_*pilA*_*-pilA*^*D71C*^ (*E*). (Scale bars: 4 μm.) (*F*) Motility phenotypic assay of strains DZ2 (WT; *Top*) and RM216 (*ΔsgmX*; *Bottom*) on soft agar plate. (*G*) Single-cell twitching motility assay of strains DZ2 (WT; black*),* TM22 (*ΔmglA*; green), RM216 (*ΔsgmX*; purple), and TM108 (*ΔpilA*; blue) observed by time-lapse phase-contrast microscopy on CMC-coated glass. Motility is measured as the mean square displacement (MSD) of single cells. The result represents the average MSD of *n* trajectories and associated SEM for each strain. (*H*) Motility phenotypic assay and EPS staining of strains EM617 (*ΔdifD/G*) and EM746 (*ΔdifD/G ΔsgmX*) on soft agar plate containing Congo Red. (*I* and *J*) TIRF microscopy images of labeled Tfpa pilin of strains RM391 (*ΔsgmX att*^*mx8*^*::P*_*pilA*_*-pilA*^*D71C*^) (*I*) and RM392 (*ΔpilB att*^*mx8*^*::P*_*pilA*_*-pilA*^*D71C*^) (*J*). (Scale bars: 4 μm.) See also Movies S8 and S9.

To elucidate how MglA-GTP recruits pilin subunits to activate S-motility, we developed a genetic screen to search for MglA-independent motility (Mim) suppressor variants that restore cell motility in the absence of MglA. To do so, we used a *M. xanthus* strain (*ΔBAR* strain hereinafter) lacking *mglA* but also lacking *mglB* (25, 26) and *romR* (24), to avoid any potential interference linked to possible moonlighting activity of the GAP/GEF system in the absence of MglA. We selected the *Mim* variants by performing standard motility assays on agar plates with the *ΔBAR* strain (*Methods*). After 2 wks of incubation at 32 °C, we observed the emergence of local motile flares of cells that escape from the otherwise nonmotile colony (*SI Appendix*, Fig. S2*A*). Selection of Mim variants was confirmed by repeating the motility assays with both the parental *ΔBAR* and a pure culture of isolated variants (*ΔBAR mim*) strain. As shown in Fig. 2*A*, the *ΔBAR* strain showed a nonmotile phenotype on both hard (both A- and S-motility) and soft (only S-motility) agar plates, while motility was restored in the *ΔBAR mimA* strain, characterized by an expansion of the cell colonies cells in both conditions (Fig. 2*A*).

We verified that only the S-motility was involved in *mim* variants by constructing a *ΔBAR mimA* strain bearing mutations inactivating the S-motility (mutations in *pilA*) or the A-motility (mutations in *aglZ* or *cglB*). In motility assays on hard agar plates where both A- and S- motility can be detected, we found in a *ΔBAR mimA* strain that only the inactivation of *pilA* could abolish cell motility, while inactivation of either *aglZ* or *cglB* did not affect motility (Fig. 2*B* and *SI Appendix*, Fig. S2*B*). Consistent with the foregoing findings, using single-cell time-lapse microscopy on hard agar surfaces, we observed motile cell groups in *ΔBAR mimA* strain—an intrinsic characteristic of the twitching-dependent motility (*SI Appendix*, Fig. S2*C* and Movie S6)—while *ΔBAR* cells did not show any motility (*SI Appendix*, Fig. S2*D* and Movie S7). We conclude that suppressor mutations in the *mim* variants selectively restore the S-motility independent of the A-motility. Thanks to the cysteine-maleimide pili labeling, we could evidence that both polar pilin clusters and pili filaments were restored in the *ΔBAR* *mim* variants (Fig. 2 *C*–*E*). Taken together, our results demonstrate that *mim* variants harbor genetic mutations that restore polar pilin subunit pools and thus polar activation of Tfpa in the absence of the normally essential MglA protein.

### SgmX, a Factor Essential for Type IVa Pili Activation.

Whole-genome sequencing of the two independent suppressor strains *ΔBAR mimA* and *ΔBAR mimB* revealed that *mim* variants harbor mutations within the same locus on *M. xanthus* chromosome (*SI Appendix*, Fig. S3*A*). The *mimA* variant has a single point mutation in the 5′ UTR of a putative operon containing four genes (Mxan_5766-63) of unknown function. More interestingly, the *mimB* variant comprised a 16-bp duplication within the *mxan_5766* gene that led to a frameshift mutation downstream of the codon encoding glycine 802, creating an altered coding sequence and introducing an early stop codon at position 859, effectively truncating the product of Mxan_5766 at the C terminus that is normally 1,060-aa long (*SI Appendix*, Fig. S3*B*). To test whether these mutations were solely responsible for the motility restoration of *mim* variants, we reintroduced *mimA* and *mimB* mutations in the parental *ΔBAR* strain (*Methods*). Both rebuilt strains *ΔBAR mxan_5766*^*mimA*^ and *ΔBAR mxan_5766*^*mimB*^ showed resumed motility compared with a *ΔBAR* strain (Fig. 2*A* and *SI Appendix*, Fig. S3*C*), showing that *mimA* and *mimB* mutations alone are sufficient to restore Tfpa function in absence of MglA.

The presence of *mim* mutations near or within the *mxan_5766* gene suggests a role of the putative Mxan_5766 protein in S-motility regulation. Supporting this, a transposon-based screen in *M. xanthus* has identified an insertion within *mxan_5766* (termed SgmX, for Social Gliding Mutant X) that impairs S-motility (34). To validate SgmX involvement in S-motility, we generated a marker-less null mutant and assessed its motility behavior. As presented in Fig. 2*F*, a *sgmX* strain was nonmotile compared with a WT strain on soft agar, a condition that permits only S-motility. The *sgmX* strain retained motility on hard agar (*SI Appendix*, Fig. S4*A*), a condition in which both A- and S- motility can be observed. However, this motility was abolished in a double *cglB sgmX* mutant strain (*SI Appendix*, Fig. S4*A*). Together, these results demonstrate that SgmX is only involved in S-motility. Further inactivation of the downstream genes *mxan_5765* or *mxan_5764* had no effect on S-motility (*SI Appendix*, Fig. S4*B*). Therefore, we conclude that SgmX is required for S-motility, and thus the *mim* mutations are likely gain-of-function mutations that bypass the need for MglA to activate motility via SgmX.

Like other *pil* mutants that inactivate pilus function, EPS production is also abolished in an *sgmX* strain, leaving the possibility that SgmX is involved in EPS synthesis (*SI Appendix*, Fig. S4*C*). While WT cells move on CMC-coated glass, supporting EPS-independent twitching (23, 33), *sgmX* mutant cells do not move in these conditions, similar to *pilA* and *mglA* mutant cells (Fig. 2*G*). Also, S-motility is not restored in a *sgmX* mutant carrying an additional deletion of the *difDG* genes (Fig. 2*H*), which is known to restore EPS production independently of Tfpa activity (35). Thus, we hypothesize that SgmX and MglA may specifically function in the same Tfpa-regulation pathway.

With the help of the cysteine-labeling method described above, we evidenced that no polar pilin clusters are formed in a *sgmX* strain, as in a *mglA* background (Fig. 2*I*). However, at variance with *mglA* cells in which sporadic assembly and activation of polar pili could be observed (Movie S4), *sgmX* cells never assembled pili filaments (Movie S8). The 50% decrease in PilA concentration in the *sgmX* strain (*SI Appendix*, Fig. S5 *A* and *B*) may account for this phenotype. To rule out this hypothesis, we expressed the PilA protein ectopically from two constitutive promoters (*Methods*). While expression from these promoters was sufficient to restore S-motility of a *pilA* strain, it did not restore S-motility of a *sgmX* strain (*SI Appendix*, Fig. S5 *C* and *D*). We conclude that although structural components of the Tfpa machineries are present within the cell, the latter are inactive in the absence of SgmX. Consistent with this conclusion, *pilB* extension motor mutant cells also show neither polar pilin clusters nor pilin filament formation (Fig. 2*J* and Movie S9). Finally, the bipolar pilin clusters and filaments are completely abolished when *mglA*^*Q82A*^ and *sgmX* mutations are combined, showing that a *sgmX* mutation is epistastic to an *mglA*^*Q82A*^ mutation (*SI Appendix*, Fig. S6 and Movie S10). Altogether, we conclude that SgmX is essential for Tfpa activation, similar to the PilB ATPase. The results further suggest that SgmX acts downstream of MglA-GTP and probably upstream of PilB, although this cannot be formerly demonstrated, because *sgmX* and *pilB* mutations show similar phenotypes in our assays.

### SgmX Dynamically Localizes at the Piliated Cell Pole Together with MglA.

**Fig. 3.**
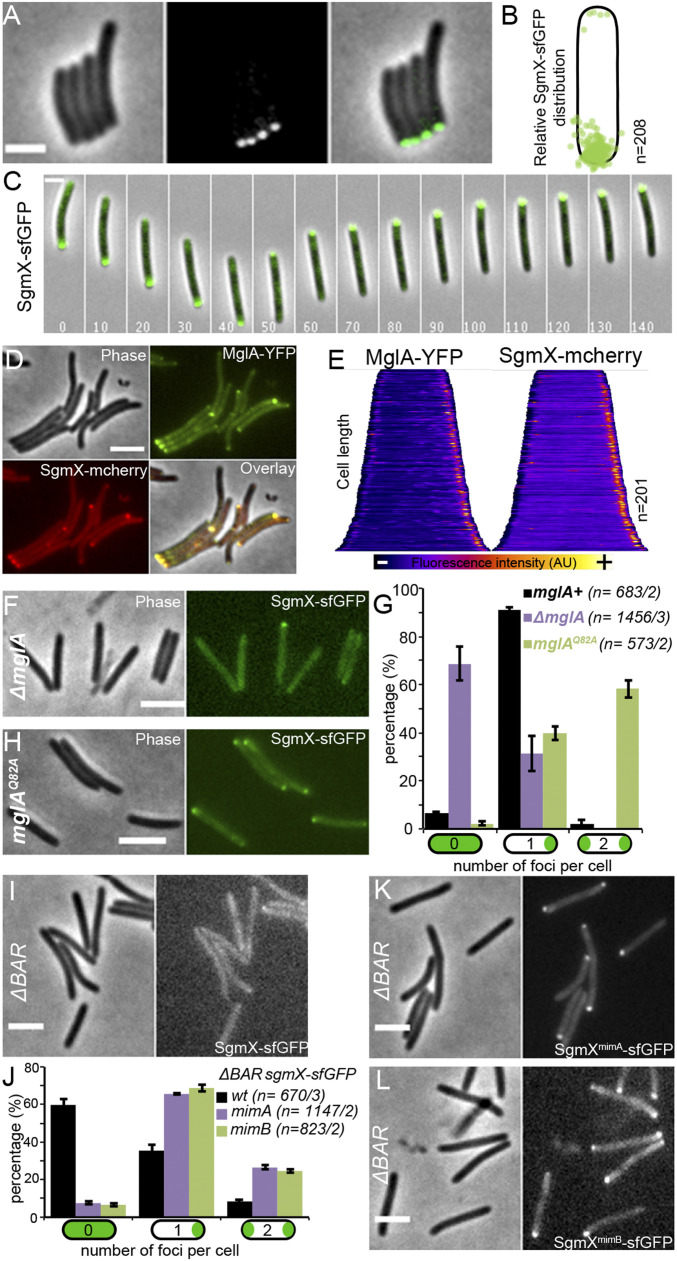
MglA-GTP regulates SgmX polar localization. (*A*) Phase-contrast (*Left*), epifluorescence (*Center*), and corresponding overlay (*Right*) images of the strain RM190 (*sgmX-sfGFP*). (Scale bar: 3 μm.) (*B*) Relative intracellular distribution of SgmX-sfGFP foci in *n* cells. (*C*) Time-lapse of pole-to-pole dynamics of SgmX-sfGFP in a single reversing RM190 (*sgmX-sfGFP*) twitching cell on CMC-coated glass. Elapsed time (s) is shown in each panel. (Scale bar: 2 μm.) See also Movie S11. (*D*) Phase-contrast (Phase), epifluorescence (MglA-YFP and SgmX-mcherry), and corresponding overlay (Overlay) images of the colocalization of SgmX-mcherry polar foci with MglA-YFP of the strain RM349 (*sgmX-mcherry mglA-YFP attMx8::P*_*mglA*_*-mglA*). (Scale bar: 3 μm.) (*E*) Corresponding whole-cell fluorescence intensity distribution of MglA-YFP (*Left*) and SgmX-mcherry (*Right*) of the strain RM349 (*sgmX-mcherry mglA-YFP attMx8::P*_*mglA*_*-mglA*) in *n* cells from *D*. Cells are organized according to cell length, and the variation of fluorescence intensity is represented by a color code at the bottom. (*F*) Phase-contrast (*Left*) and corresponding epifluorescence (*Right*) images of the strain RM194 (*ΔmglA sgmX-sfGFP*). (Scale bar: 3 μm.) (*G*) Histogram representing the proportion of cells with zero, one, or two SgmX-sfGFP foci per cell in strains RM190 (*sgmX-sfGFP; black*), RM194 (*ΔmglA sgmX-sfGFP;* purple), and RM353 (*mglA*^*Q82A*^ *sgmX-sfGFP;* green). The result represents the average proportion of *n* cells of at least two independent experiments and associated SD of the mean. (*H*) Phase-contrast (*Left*) and corresponding epifluorescence (*Right*) images of the strain RM353 (*mglA*^*Q82A*^ *sgmX-sfGFP*). (Scale bar: 3 μm.) (*I*) Phase-contrast (*Left*) and corresponding epifluorescence (*Right*) images of the strain RM275 (*ΔBAR sgmX-sfGFP*). (Scale bar: 3 μm.) (*J*) Histogram representing the proportion of cells with zero, one, or two SgmX-sfGFP foci per cell in strains RM275 (*ΔBAR; black*), RM192 (*ΔBAR*^*mimA*^ *sgmX-sfGFP;* purple), and RM260 (*ΔBAR*^*mimB*^ *sgmX-sfGFP;* green). The result represents the average proportion of *n* cells of at least two independent experiments and associated SD of the mean. (*K* and *L*) Phase-contrast (*Left*) and corresponding epifluorescence (*Right*) images of strain RM192 (*ΔBAR*^*mimA*^ *sgmX-sfGFP*) (*K*) and RM260 (*ΔBAR*^*mimB*^ *sgmX-sfGFP*) (*L*). (Scale bars: 3 μm.)

We constructed SgmX-sfGFP and SgmX-mCherry fusion proteins to investigate SgmX intracellular localization. In the corresponding strains, *sgmX* fusions are expressed from the original *sgmX* locus in the absence of antibiotic markers. SgmX-sfGFP– and SgmX-mCherry–expressing cells were S-motile, albeit with only a minor motility reduction compared with the WT cells, showing that these fusions are mostly functional (*SI Appendix*, Fig. S7*A*). Western blot analysis confirmed that the fusion protein was fully stable in vivo (*SI Appendix*, Fig. S7*B*). Fluorescent microscopy revealed that SgmX-sfGFP formed predominantly unipolar clusters in cells (Fig. 3 *A* and *B*). During twitching motility on CMC, SgmX-sfGFP localized at the leading cell pole, and this localization switched poles when cells reversed (Fig. 3*C* and Movie S11). As expected, SgmX-mCherry and MglA-YFP colocalized when dually expressed. In the latter case, MglA-YFP is expressed in the presence of WT MglA, since MglA-YFP is only partially functional (26). We conclude that SgmX localizes at the piliated cell pole, where it could activate Tfpa, together with MglA.

### MglA-Dependent SgmX Polar Localization Activates Tfpa.

We searched for factors that could mediate SgmX polar localization. We first demonstrated that the Tfpa machinery itself is not involved in SgmX polar localization (*SI Appendix*, Fig. S8). Indeed, the polar distribution of SgmX in a WT strain is very close to that in a mutant that does not express the major Tfpa platform OM-secretin PilQ protein (*SI Appendix*, Fig. S8) (6). Second, we observed that MglA is directly required for SgmX-polar localization; while in a WT strain, a vast majority of cells exhibited a unipolar distribution pattern (90%), SgmX-sfGFP was diffuse in ∼70% of *mglA* cells, with only ∼30% of the cells retaining a unipolar pattern (Fig. 3 *F* and *G*). Thus, MglA seems to be an important factor for SgmX intracellular localization, although it is not absolutely required. Consistent with MglA acting as a SgmX polar targeting factor, SgmX-sfGFP localized at both cell poles in *mglA*^*Q82A*^ cells, which explains the bipolar activation of Tfpa (Fig. 3 *G* and *H*). Therefore, MglA mediates SgmX polar localization, which mediates Tfpa machine activation.

We next investigated how gain-of-function *sgmX* mutations restored motility in strains lacking *mglA*. In a *ΔBAR* background, SgmX-sfGFP is mostly diffuse in cells, although the amount of protein is similar to that in WT cells, suggesting that the motility defect is due to SgmX-sfGFP mislocalization (Fig. 3 *I* and *J* and *SI Appendix*, Fig. S7 *B* and *C*). We hypothesize that *mim* mutations could restore motility if SgmX polar localization were maintained in the absence of MglA. To test this, we constructed *ΔBAR* strains expressing *sgmX-sfGFP* but also carrying the *mimA* or *mimB* mutations (thus generating *mimA* SgmX-sfGFP and SgmX^*mimB*^*-sfGFP*) (*Methods*). In both cases, motility was restored along with the polar localization of SgmX-sfGFP (Fig. 3 *J*–*L* and *SI Appendix*, Fig. S7*D*). We conclude that polar pilin cluster formation is linked to SgmX polar localization (Fig. 2 *E* and *F*).

Thus, the suppressor mutations render SgmX localization independent of MglA. Based on these results, we deduce that in WT, MglA-GTP activates Tfpa machines by targeting the essential SgmX Tfpa machine activator to the cell pole.

### MglA Regulates Asymmetric Polar Localization of SgmX by Direct Interaction.

**Fig. 4.**
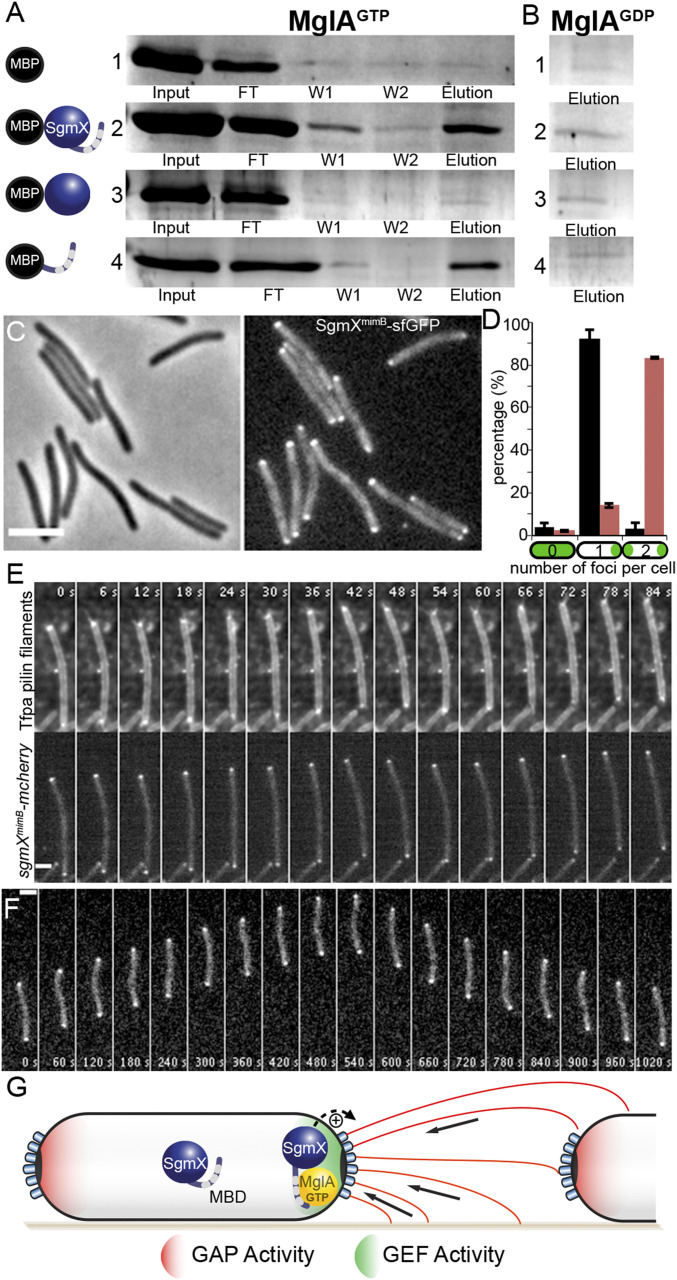
Direct interaction of MglA-GTP with SgmX-C_Ter_ regulates asymmetric polar SgmX distribution. (*A* and *B*) Pull-down experiment of purified MglA-6His preincubated with GTP (*A*) or GDP (*B*) with purified MalE (1), MalE-SgmX^D2-L1060^ (2), MalE-SgmX^D2-D853^ (3), or MalE-SgmX^A813-L1060^ (4) bound to amylose resin. The different lanes represent the following: input, the purified MglA loaded on the column; flow-through (FT), the unbound MglA; wash (W1, W2), washes with buffer; and elution, MglA bound to SgmX. Samples were migrated on SDS-PAGE, and protein bands were revealed by Coomassie blue staining. (*C*) Phase- contrast (*Left*) and corresponding epifluorescence (*Right*) images of the strain RM288 (*sgmX*^*mimB*^*-sfGFP*). (Scale bar: 3 μm.) (*D*) Histogram representing the proportion of cells with zero, one, or two SgmX-sfGFP (black) or SgmX^mimB^-sfGFP (red) foci per cell of strains RM190 (*sgmX-sfGFP; n = 214*) or RM288 (*sgmX*^*mimB*^*-sfGFP; n = 686*), respectively. The result represents the average proportion of n cells of two independent experiments and associated SD of the mean. (*E*) Time-lapse series of Tfpa pilin filaments and polar cluster enrichment (*Top*) and of SgmX^mimB^-mcherry foci (*Bottom*) of a cell of the strain RM393 (*sgmX*^*mimB*^*-mcherry att*^*mx8*^*::P*_*pilA*_*-pilA*^*D71C*^) observed by TIRF microscopy. Elapsed time (s) is shown in each panel. (Scale bar: 2 μm.) See also Movies S12 and S13. (*F*) Time-lapse series of SgmX^mimB^-sfGFP foci dynamics in a single reversing RM288 (*sgmX*^*mimB*^*-sfGFP*) cell on agar pad 1.5%. Elapsed time (s) is shown in each panel. (Scale bar, 2 μm.) See also Movie S14. (*G*) A model for Tfpa activation by the small GTPase MglA in *M. xanthus*. MglA-GTP is asymmetrically localized at the leading pole of the cell (by the combined action of MglB (GAP Activity, Red) and RomR/X (GEF Activity, Green). The SgmX-C_Ter_-TPR Domain (Mgla Binding Domain) inhibits SgmX polar localization. Thus, the interaction of between MglA-GTP and the SgmX C_Ter_-TPR Domain unmasks a polar binding site that tether SgmX to the pole where it activates Tfpa machines.

In absence of MglA, SgmX can still localize to the pole, albeit with severely reduced efficiency, suggesting that SgmX bears a polar localization motif that becomes more efficiently exposed in the presence of MglA. This hypothesis suggests that MglA directly interacts with SgmX. To explore this possibility, we performed an in vitro pull-down experiment with purified N-terminal MalE-tagged SgmX (MalE-SgmX) and C-terminal His_6_-tagged MglA (MglA-His_6_). MglA-His_6_ was preincubated with either GDP or GTP and then mixed with MalE and MalE-SgmX proteins preimmobilized on amylose resin. We found that MglA-GTP could specifically interact with MalE-SgmX, as we retrieved both proteins from the eluate (Fig. 4*A*). This interaction is specific and occurs only for the MglA-GTP active form, for the following reasons: 1) we retrieved no MglA-GTP in the elution fraction when incubated with MalE alone, confirming that the interaction observed is SgmX-specific (Fig. 4*A*); and 2) MglA-GDP was not recovered in the elution fraction (only in the washes) when it was incubated with MalE-SgmX (Fig. 4*B* and *SI Appendix*, Fig. S9*A*).

The SgmX protein is composed solely of 14 tetratricopeptide repeats (TPRs) (*SI Appendix* and Fig. 3*B*), a well-known protein–protein interaction structural motif (36, 37). Of note, a TPR domain functional unit is commonly composed of three TPR repeats (37). Remarkably, the gain-of-function SgmX^mimB^ variant, which bypasses the requirement of MglA for SgmX polar localization and twitching motility activation, lacks the last three C-terminal TPR repeats (TPRs12-14; Fig. 3*B*). Therefore, we speculated that SgmX TPRs12-14 could form a TPR motif that interacts with MglA. To test this, we purified a MalE protein fused to SgmX lacking this domain (MalE-SgmX^ΔTPR12-14^) and a MalE protein fused to this domain alone (MalE-TPR12-14). MalE-SgmX^ΔTPR12-14^ did not interact with MglA-GTP or MglA-GDP (Fig. 4 *A* and *B* and *SI Appendix*, Fig. S9*A*). In contrast, and similar to the full-length MalE-SgmX protein, the MalE-TPR12-14 protein interacted with MglA-GTP but not with MglA-GDP (Fig. 4 *A* and *B* and *SI Appendix*, Fig. S9*A*).

Taken together, these results suggest that MglA-GTP binds to the C-terminal domain of SgmX, unmasking a polar binding site in the SgmX protein. To reconcile this with our observations in the SgmX^mimB^ variant, we hypothesize that constitutive exposure of the polar binding site would bypass the requirement for MglA for localization. To go further, we examined the localization profile of SgmX^mimB^-GFP variant (in the absence of the WT SgmX) in an otherwise WT strain (MglA^+^). In this background, SgmX^mimB^-sfGFP was mostly bipolar (Fig. 4 *C* and *D*). Importantly, the bipolar localization of SgmX^mimB^ correlated with the bipolar activation of Tfpa machines (Fig. 4*E* and Movies S12 and S13). Moreover, although S-motility proteins (i.e., SgmX itself and FrzS) oscillated in a coordinated fashion with A-motility proteins during motility reversals on 1.5% agar (where motility is driven by the Agl/Glt complex and not by Tfpa), SgmX^mimB^-sfGFP no longer oscillated and remained static at both poles of the cell (Fig. 4*F* and Movie S14). We conclude that the interaction between MglA-GTP and SgmX allows for regulation of SgmX localization, targeting it to one cell pole and allowing Tfpa reversals when MglA-GTP is switched to the opposite pole. This regulation is essential because even though the *mimB* mutant is motile on agar, its colony expansion capacity is strongly reduced compared with the WT strain (*SI Appendix*, Fig. S9*B*), an effect that is known for reversal mutants (17). Finally, these observations also explain the *mimA* suppressor effect. Indeed, we observed that this mutation, situated upstream of the *sgmX* ATG initiation codon, leads to SgmX overexpression (*SI Appendix*, Fig. S10). Thus for the *mimA* variant, a mass action effect likely restores SgmX polar localization in the absence of MglA.

## Discussion

### Motility Regulation by the Small GTPase MglA and Cell Polarity in *M. xanthus*.

In *M. xanthus* cells, MglA-GTP is asymmetrically localized at the leading pole of the cell owing to the combined action of MglB and RomR/X (Fig. 4*G*). Remarkably, MglA-GTP can selectively interact with a number of effectors depending on the conditions; it can recruit MreB/AglZ to activate A-motility (29, 30) or the newly identified SgmX protein to activate S-motility. Several lines of evidence suggest that SgmX is the major MglA effector that mediates pole-specific activation of the S-motility and thus MglA-dependent pole-switching of Tfpa machine activity: 1) in the absence of *mglA*, polar pili are assembled, but they can emerge occasionally at both cell poles, showing that MglA is not strictly required for Tfpa machines activity but is required for their efficient, coordinated activation at one cell pole only; 2) MglA-dependent SgmX polar localization correlates with the polar activation of Tfpa machines—for example, the bipolar activation of Tfpa machines is greatly increased in the presence of an MglA^Q82A^ mutation (no GDP hydrolysis), which recruits SgmX at both cell poles; 3) a SgmX^mimB^ variant that conserves a polar localization in absence of MglA restores polar activation of Tfpa machines and twitching motility; and 4) polarity regulation is abolished in an MglA^+^ strain, leading to bipolar SgmX^mimB^ localization that results in bipolar activation of Tfpa machines.

Thus, MglA is required for targeting SgmX to cell poles, which in turn activates Tfpa (see below) in a mechanism that seems to be dependent on dynamic changes in the SgmX conformational state. We hypothesize that SgmX conformational switching is controlled by the SgmX TPR C-terminal domain, which would occlude a polar binding motif when SgmX does not interact with MglA (Fig. 4*G*).

Remarkably, the suppressor analysis revealed that cell polarity can be established in *M. xanthus* independent of the MglAB-RomRX system because SgmX^mimA^ or SgmX^mimB^ are nevertheless asymmetrically distributed in a majority of BAR cells (Fig. 3 *J*–*L*). Thus, another mechanism of polarization must exist, perhaps inherited from cell division to explain the polarization of SgmX to the pole. Nevertheless, the MglAB-RomR system is essential for switching this polarity and creating a dynamic polarity axis.

### Function of SgmX in Twitching Motility Activation.

#### Tfpa activation.

Our results led us to establish that the C terminus TPR domains of SgmX mediate regulation but not function; polar-localization and Tfpa activation are likely encoded upstream of this motif, possibly separately, as the protein contains no fewer than 11 additional TPR domains. The polar localization mechanism remains to be determined, but it does not require Tfpa machine assembly (*SI Appendix*, Fig. S8). Regarding Tfpa activation, given that we observed similar phenotypes in both *sgmX* and *pilB* strains (i.e., with the abolition of pilus filament assembly; Fig. 2), PilB function is abolished in absence of SgmX, suggesting that SgmX plays a role in activation of PilB function (directly or indirectly).

#### Tfpa coordination.

We directly observed that twitching cells coordinate extension-retraction cycles of multiple Tfpa filaments at the pole, which possibly involves several Tfpa machineries, as the cell poles contain up to 10 distinct complexes (Fig. 1*A* and Movie S1) (6). In bacterial cells, so-called “hub” proteins assemble at cell poles and coordinate cellular processes as diverse as flagellar motility, signal transduction, development, polar organelle synthesis, chromosome replication, and cell division (i.e., HubP, PodJ, and DivIVA) (38–41). A hallmark of these hub proteins is the higher-order oligomers assembly forming polar meshworks. It will be interesting to test whether this also a property of SgmX, which could link the activity of multiple Tfpa to their regulation by signal transduction and environmental signals and also perhaps via MglA and the Frz-signaling pathway.

### Tfpa Regulations in Other Bacteria.

SgmX-like proteins may also regulate Tfpa function in other bacteria. A remote SgmX-homolog (Bd2492) is also found in *Bdellovibrio bacteriovorus* and is essential for predation (42). Remarkably, Bd2492 could be pulled down with the *Bdellovibrio* MglA homolog (although the exact nucleotide dependence and interaction motifs were not investigated), and both proteins were proposed to be part of a polar hub for prey invasion. Involvement of Bd2492 in Tfpa function was not tested, but we hypothesize that it is also involved in Tfpa function, which would explain why it is required for interaction with prey cells (43–45).

More generally, the cysteine-labeling pilin method that we used here to visualize pilin filaments dynamics in twitching cells confirms that pilin filaments essentially work as retractile grappling hooks to propel cells (12, 13), and also shows that periplasmic pilin accumulates locally when Tfpa machines are activated. The formation of this “pool” is not required for pilus elongation per se; although this pool is not present in *mglA* mutants, Tfpa filaments can still be observed. Consistent with this, cysteine-labeled *Caulobacter crescentus* Tad and *Vibrio cholerae* competence pilins do not accumulate at the poles, but rather localize peripherally along the membrane and can still be mobilized for pilus elongation (31, 32). Thus, we propose that polar pools form as multiple Tfpa basal bodies are activated at the cell pole during twitching motility. At the molecular level, we have determined that polar pools require SgmX and PilB. Remarkably, polar pools are present in all cells irrespective of whether they are actively elongating pili or not; this observation reveals that PilB can mediate pilin recruitment without inducing pilin filament extension (Movie S3). This is an important observation, because the exact nature of the dynamic regulation occurring between PilB and PilT to enable Tfpa filament synthase/retraction remains poorly understood. Other twitching bacteria likely coordinate Tfpa at the cell pole, which could also involve polar pilin pools at the active cell pole controlled by PilB and possibly SgmX-like assemblages. The development of cysteine-labeling pilin method in other twitching bacteria will be key to addressing this question.

### Materials and Methods.

Detailed descriptions of bacterial strains, plasmids, and growth conditions; protein purification and pull-down assays; Western blot analysis; and microscopy and image analysis are provided in *SI Appendix*.

### Type IV Pilus Labeling and Observation.

For type IV pili filament labeling, *M. xanthus* strains carrying the plasmid pSWU19-*P*_*pilA*_*-pilA*^*D71C*^ were grown in CYE medium until midexponential phase. Cells were injected in a preassembled Ibidi sticky-Slide VI 0.4 microfluidic device sealed with a glass slide, coated with 0.015% carboxymethylcellulose (33). After 30 min of incubation, Alexa Fluor 488 dye (Invitrogen) was added at 20 μg/mL in TPM buffer with 1 mM CaCl_2_ for 10 min in the dark, and cells were washed several times with TPM buffer with 1 mM CaCl_2_. Cells were imaged on a DeltaVision OMX SR Imaging system (GE Healthcare) in total internal reflection fluorescence (TIRF) mode with a 60× 1.49 NA TIRF objective and laser illumination (IMM Microscopy Platform).

Pictures and movies were prepared for publication using Fiji (https://fiji.sc/) and Adobe Photoshop.

### Selection of Mutations Promoting MglA-Independent Motility and Motility Phenotypic Assay.

For selection of *ΔBAR*^*mimA*^ and *ΔBAR*^*mimB*^ strains, a nonmotile *ΔBAR* strain was grown in CYE medium until midexponential phase, and cells were concentrated to an OD_600_ of 5 in TPM buffer (10 mM Tris⋅HCl pH 7.6, 8 mM MgSO_4_, and 1 mM KH_2_PO_4_). Then cells were spotted (5 μL) on CYE 1.5% agar plates and incubated at 32 °C for 2 wks, until motile flares emerged from the colony. Flares of *ΔBAR*^*mimA*^ and *ΔBAR*^*mimB*^ strains were selected and their genomic DNA was extracted, and mutations were identified by whole-genome sequencing.

For motility phenotypic assays, exponentially growing cells in CYE medium at 32 °C were adjusted to an OD_600_ of 5 in TPM buffer and spotted (5 μL) on CYE plates containing an agar concentration of 0.5% (soft) or 1.5% (hard), incubated at 32 °C, and photographed after 48 h with an Olympus SZ61 or Nikon Eclipse TE2000E microscope.

### Genome Sequencing and Identification of SNPs.

Whole-genome sequencing was performed with the Illumina MiSeq System at the IMM Transcriptomic Platform. Sequencing samples were prepared using the Illumina Nextera XT DNA Library Preparation Kit according to the manufacturer’s instructions. Sequence reads were aligned with Unipro Ugene software (46) using the NCBI *M. xanthus* DK1622 genome (GenBank assembly accession no. GCA_000012685.1) as a reference. SNPs and nucleotide deletions/insertions were analyzed with Unipro Ugene software (46). Genetic variations were confirmed by Sanger sequencing (Eurofins GATC-Biotech).

## Supplementary Material

Supplementary File

Supplementary File

Supplementary File

Supplementary File

Supplementary File

Supplementary File

Supplementary File

Supplementary File

Supplementary File

Supplementary File

Supplementary File

Supplementary File

Supplementary File

Supplementary File

Supplementary File

## Data Availability

All data supporting this study are included in the main text and *SI Appendix*.
