# Supplementary material for: The polar Ras-like GTPase MglA activates type IV pilus via SgmX to enable twitching motility in Myxococcus xanthus
Source: Proc Natl Acad Sci U S A. 2020 Oct 22;117(45):28366–73. doi: 10.1073/pnas.2002783117 (PMC7668184; doi:10.1073/pnas.2002783117)
Supplement: Supplementary File [file pnas.2002783117.sapp.pdf]

PNAS
www.pnas.org

Supplementary Information for

The polar Ras-like GTPase MglA activates type IV pilus via SgmX to enable Twitching motility in *Myxococcus xanthus.*

Romain Mercier, Sarah Bautista, Maëlle Delannoy, Margaux Gibert, Annick Guiseppi, Julien Herrou, Emillia M. F Mauriello and Tâm Mignot

Correspondence to rmercier@imm.cnrs.fr or tmignot@imm.cnrs.fr

This PDF file includes:

Figs. S1 to S10
Tables S1 and S2
Legends for Movies S1 to S14
References for SI reference citations

Other supplementary materials for this manuscript include the following:

Movies S1 to S14

www.pnas.org/cgi/doi/10.1073/pnas.2002783117

Supplementary Materials & Methods

(I) Bacterial Strains, Plasmids, and Growth Conditions

Strains, Plasmids, and Growth Conditions. The bacterial strains and plasmid constructs used in this study are shown in SI Appendix, Tables S1-2. DNA manipulations and *E. coli* DH5α transformation were carried out using standard methods (1). All plasmid constructions were verified by Sanger sequencing (Eurofins GATC-Biotech, Germany). Plasmids were introduced in *M. xanthus* by electroporation. Mutants and expression of protein fusions were obtained by integration-excision recombination method as previously reported (2) or by site-specific integration at the Mx8-phage attachment site (2). *E. coli* cells were grown in Luria-Bertani broth (LB) and on Luria-Bertani 1,5% Agar plate. *M. xanthus* DZ2 or DK1622 cells were grown in CYE media (1% (w/v) Casitone, 0.5% yeast extract, 10 mM MOPS (pH 7.6) and 4 mM $MgSO_4$) and on CYE 1,5% or 0,5% Agar plates at 32°C. For EPS staining on plate, the Congo red was used at a concentration of 30 µg/ml. When necessary, the following antibiotics were added to media at the indicated concentrations: kanamycin, 50 µg/ml or 200 µg/ml; Ampicillin, 100 µg/ml; Tetracycline, 5 µg/ml or 10 µg/ml.

Strains and plasmids constructions. *mimA* and *mimB* mutations were reintroduced into the parental strain TM500 (*ΔBAR*) to respectively create the strains RM310 (*ΔBAR* $mxan\_5766^{mimA}$) and RM244 (*ΔBAR* $mxan\_5766^{mimA}$). To do so, the plasmids pBJ114-$mxan\_5766^{mimA}$ and pBJ114-*sgmX-sfGFP* were transformed into the strains RM55 (*ΔBAR mimA*) and RM77 (*ΔBAR mimB*), respectively. Genomic DNA of the two transformed strains was isolated and used to transform the parental strain TM500 (*ΔBAR*). Sanger sequencing prior the excision of the plasmid confirmed the transfer of *mimA* or *mimB* mutations.

Plasmids pBJ114 carrying *sgmX-sfGFP* and *sgmX-mcherry* or $sgmX^{mimB}$-*sfGFP* and $sgmX^{mimB}$-*mcherry* were constructed by Gibson assembly of DNA fragments allowing to fuse, at the locus on the chromosome, *sgmX* or $sgmX^{M1\text{-}A809}$ in frame with the *sfGFP* or *mcherry* genes, respectively.

Plasmids pSWU19 carrying P_{mxan_3192}-*pilA* and P_{mxan_1254}-*pilA* were constructed by Gibson assembly of DNA fragments containing 1000 bp upstream of higly expressed genes *mxan_3192* or *mxan_1254* (3) and *pilA* gene. Plasmids were integrated on the chromosome at Mx8 phage *attB* site by site specific recombination.

Protein expression plasmids pMal-c2G carrying *sgmX*, $sgmX^{\Delta MBD}$ and $sgmX^{MBD}$ were constructed by Gibson assembly allowing to fuse in frame respectively $sgmX^{D2\text{-}L1060}$ (MalE-SgmX), $sgmX^{D2\text{-}D853}$ (MalE-$SgmX^{\Delta TPR12\text{-}14}$) and $sgmX^{A813\text{-}L1060}$ (MalE-TPR12-14) at the C-terminal of *malE* gene.

(II) Proteins Purification and Pull-Down assay.

Proteins Purification. All proteins were expressed in *E. coli* BL21(DE3)pLysS strain grown in LB medium. Briefly, cells were grown at 37°C until mid-exponential phase. Protein expression was induced by addition of 1mM IPTG for 4h at 30 °C. Cells were pelleted and stored at -80°C.

To purified MglA-His, a pellet of MglA-His expressing cells were resuspended in lysis buffer (BugBuster® Millipore) complemented with Dnase I and a protease inhibitor cocktails (cOmplete™ Roche). Lysate was clarified by centrifugation and was loaded onto a gravity

column prepacked with HisPur^{Tm} Ni-NTA Resin (Thermoscientific) equilibrated with a buffer containing 10 mM Tris (pH7.4), 150 mM NaCl, 10 mM Imidazole, 10 mM $MgCl_2$. After 10 min of incubation, the resin was washed with 4-column volume (40 ml) of a buffer containing 10 mM Tris (pH7.4), 150 mM NaCl, 75 mM Imidazole, 10 mM $MgCl_2$ and MglA-His protein was eluted with a buffer containing 10 mM Tris (pH7.4), 150 mM NaCl, 500 mM Imidazole, 10 mM $MgCl_2$. Protein purity was assessed by SDS–polyacrylamide gel and revealed by Coomassie blue staining; protein concentration was measured by Nanodrop. Purified MglA-His protein was directly incubated with 80 µM of GTP or GDP for 25 minutes at 4 °C and processed for pull-down assay experiments.

Pull-Down assay. For pull-down assay with purified MglA-His protein, MBP, MBP-SgmX, MalE-SgmX$^{\Delta TPR12\text{-}14}$ and MalE-TPR12-14, pellets of the protein expression strains expressing the corresponding recombinant proteins were resuspended in lysis buffer (BugBuster® Millipore) complemented with Dnase I and a protease inhibitor cocktails (cOmplete^{Tm} Roche). Lysates were clarified by centrifugation and were loaded onto a gravity column prepacked with Amylose Resin (BioLabs) equilibrated with a buffer containing 10 mM Tris (pH7.4), 150 mM NaCl, 10 mM Imidazole and 10 mM $MgCl_2$. After 10 min of incubation, resin were washed with 4-column volume (40 ml) with a buffer containing 10mM Tris (pH7.4), 150 mM NaCl and 10 mM $MgCl_2$ and 1-column volume (10 ml) with a buffer containing 10 mM Tris (pH7.4), 150 mM NaCl, 10 mM $MgCl_2$ and 80 µM GDP or GTP. MglA^{GTP}-His or MglA^{GDP}-His were then loaded onto MBP, MBP-SgmX, MalE-SgmX$^{\Delta TPR12\text{-}14}$ and MalE-TPR12-14 fixed amylose resins, respectively pre-equilibated with GTP or GDP. After 10 min of incubation, amylose resins were washed twice with 1ml with a buffer containing 10 mM Tris (pH7.4), 150 mM NaCl, 10 mM $MgCl_2$ and 80 µM GDP or GTP and eluted by addition of 200 µl of a buffer containing 10 mM Tris (pH7.4), 150 mM NaCl, 10 mM $MgCl_2$ and 50 mM Maltose. Protein samples (20µl) were loaded on 10% SDS–polyacrylamide gels at 180 V for 60 min and protein bands were revealed by standard Coomassie blue staining.

(III) Western blots.

Samples were grown at 32 °C in CYE medium until mid-exponential phase. Cells were adjusted to OD_{600nm} of 10 in 2x SDS–PAGE loading buffer containing β-mercaptoethanol and heated for 10 min at 99 °C. Protein samples (10 µl) were separated on 10% SDS–polyacrylamide gel at 180 V for 60 min at room temperature. For western blotting, proteins were transferred from the gels onto nitrocellulose membranes. The membranes were blocked for 1 h at room temperature in PBS (pH 7.6), 5% milk and 0.2% Tween 20 (α-GFP and α-PilA) or in TBS (pH 7.6), 5% milk and 0.05% Tween 20 (α-MglA and α-CglB) and incubated with primary antibodies directed against GFP, MglA, PilA or CglB diluted at 1:10,000 in their respective blocking buffer overnight at 4 °C. After three 5-min washes with PBS (pH 7.6), 5% milk and 0.2% Tween 20, the membranes were incubated with goat anti-rabbit IgG (H + L)-HRP conjugate (1706515, Bio-Rad). The peroxidase reaction was developed by chemiluminescence (SuperSignal West Pico Chemiluminescent Substrate; 34080, Thermo Scientific), scanned with ImageQuant LAS 4000 and with analysed Fiji (https://fiji.sc/).

(IV) Microscopy and Image Analysis.

For standard microscopy, exponentially growing cells grown in CYE media were washed, concentrated in TPM buffer and mounted on microscope slides covered with an 1.5% TPM

agarose pad. The cells were imaged on an automated and inverted epifluorescence microscope TE2000-E-PFS (Nikon), with a ×100/1.4 DLL objective and a camera orca flash 4 (Hamamatsu) at room temperature. Mercury fluorescent lamp with Green and Red optical filters was used when necessary.

For single cell twitching microscopy, cells grown in CYE media until OD_{600nm} of 0.3 were directly injected in a preassemble Ibidi sticky-slide VI$^{0.4}$ (Ibidi) microfluidic devise sealed with a glass slide, coated with 0.015% carboxymethylcellulose (4). Cells were incubated for 30 min and washed several times with TPM buffer with 1 mM $CaCl_2$. The cells were imaged on an automated and inverted epifluorescence microscope TE2000-E-PFS (Nikon), with a ×100/1.4 DLL objective and a camera orca flash 4 (Hamamatsu) at room temperature. Mercury fluorescent lamp with Green and Red optical filters was used when necessary.

Images analysis were performed using Fiji plugins MicrobeJ (5) and cell Counter. Pictures and movies were prepared for publication using Fiji (https://fiji.sc/) and Adobe Photoshop.

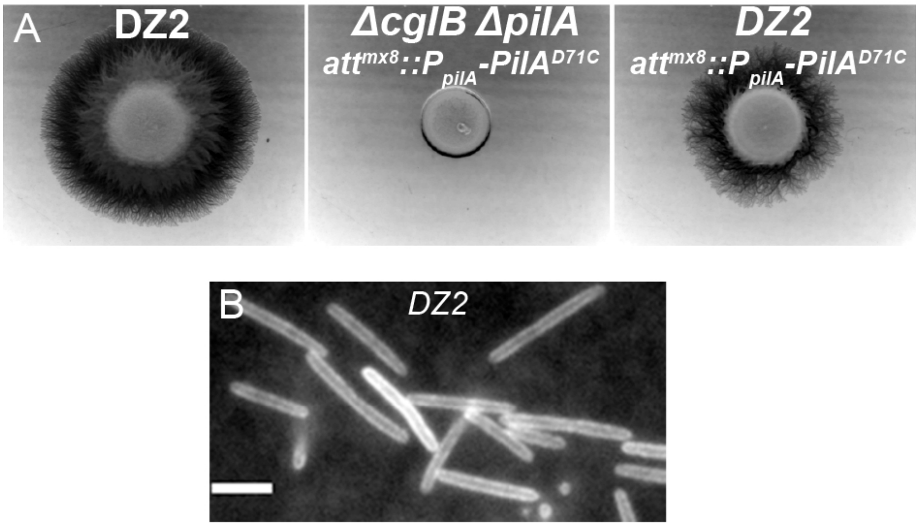

Fig. S1. Merodiploid PilA^{D71C} and PilA^{wt} proteins expression allows motility resumption. (A) Motility phenotypic assay of strains DZ2 (*Wild type*; left), RM382 (*ΔcglB ΔpilA att^{mx8}::P$_{pilA}$-pilA^{D71C};* center) *and* RM384 (DZ2 *att^{mx8}::P$_{pilA}$-pilA^{D71C};* right) on soft agar plate. (B) TIRF microscopy images of labelled Tfpa pilin of the strain DZ2 (*Wild type*). Scale bar: 4 µm.

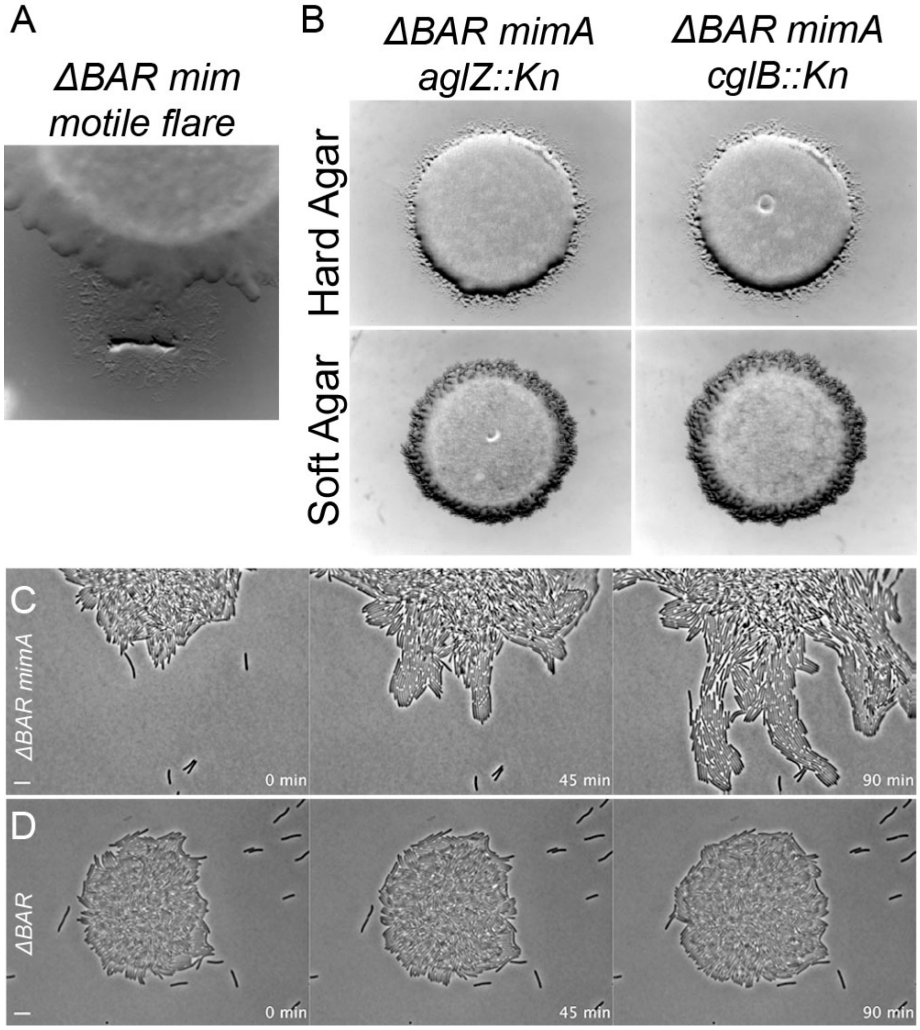

Fig. S2. Characterisation of Mgla-independent motility (Mim) variants. (A) Example of a motile flare representing *mim* variant cells escaping from the parental non-motile strain TM500 (*ΔBAR)* colony. (B) Motility phenotypic assay of strains RM375 (*ΔBAR^{mimA} aglZ::Kn;* left*)* and RM98 (*ΔBAR^{mimA} cglB::Kn;* right) on hard (top) and soft (bottom) agar plates. (C-D) Cell motility of RM55 *(ΔBAR mimA*, C) and TM500 (*ΔBAR*, D*)* strains observed by time-lapse phase contrast microscopy. Elapsed time (min) is shown in each panel. Scale bars: 3 µm. See also SI Appendix, Movies S6-7.

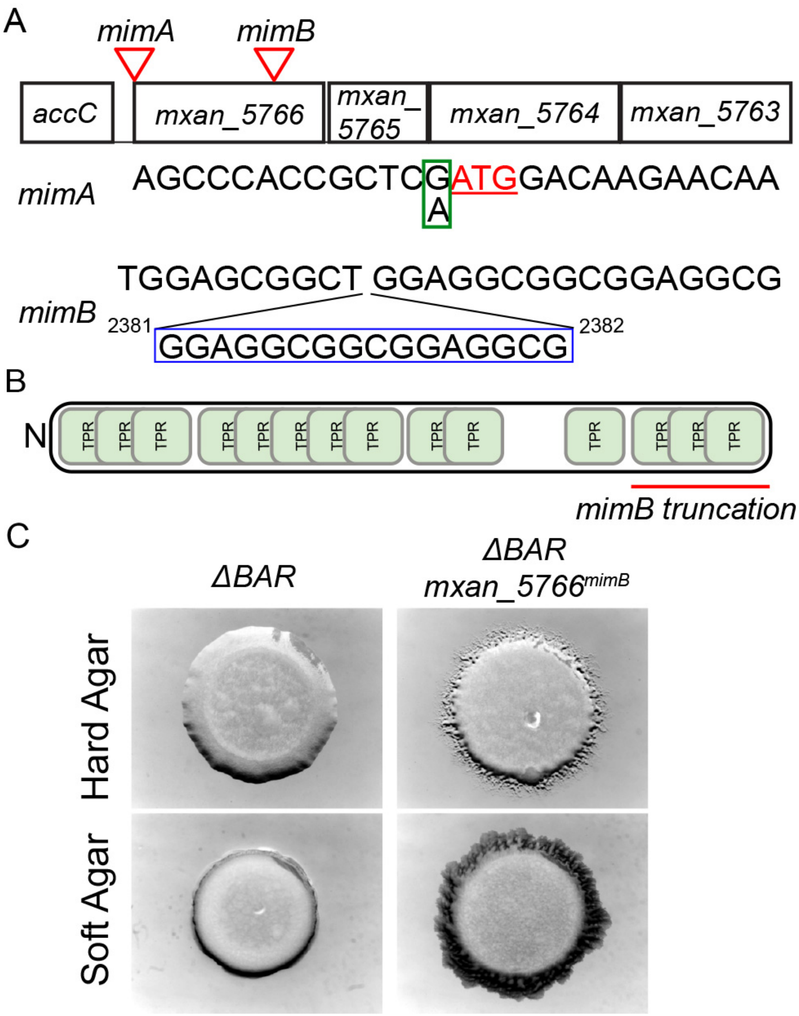

Fig. S3. Mim mutations located in the gene *mxan_5766* are sufficient to restores the motility of a *ΔBAR* strain. (A) Schematic representation of the *M. xanthus* genomic region containing the *mxan_5766-63* genes. The position of *mim* mutations is represented by red triangle. The G→A substitution corresponding to the *mimA* mutation is shown by a green square and the start codon of Mxan_5766 (ATG) in red. The 16 pb insertion corresponding to the *mimB* mutation is shown by a blue square and the position of the insertion by black bar. (B) Schematic representation of the *M. xanthus* Mxan_5766 protein. The 14 tetratricopeptide repeats (TPR) characterised using TPRpred (https://toolkit.tuebingen.mpg.de(6)) are represented by green squares. The red line represents the truncation of Mxan_5766 obtained in the *mimB* variant. (C) Motility phenotypic assay of strains TM500 (*ΔBAR;* left*)* and RM244 (*ΔBAR mxan_5766^{mimB}*, right) on hard (top) and soft (bottom) agar plates.

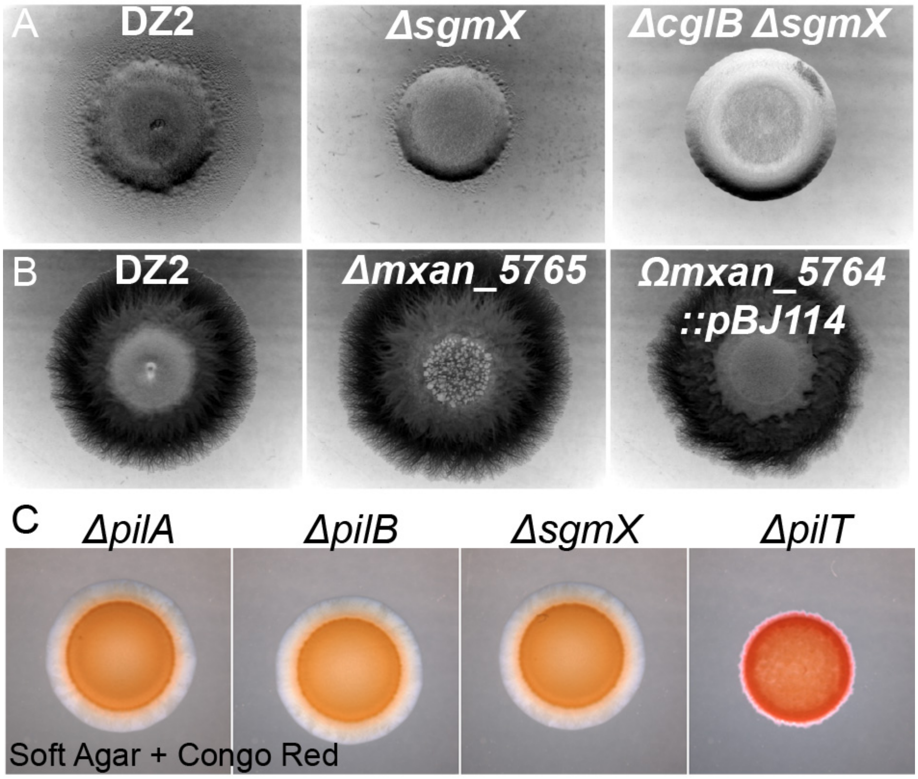

Fig. S4. SgmX is essential for S-motility and EPS synthesis. (A) Motility phenotypic assay of strains DZ2 (*Wild type;* left), RM216 (*ΔsgmX;* center) and RM182 (*ΔcglB ΔsgmX;* right) on hard agar plate. (B) Motility phenotypic assay of strains DZ2 (*Wild type;* left), RM185 (*Δmxan_5765;* center) and RM187 (Ω*mxan_5764::pBJ114;* right) on soft agar plate. (C) Motility phenotypic assay and EPS staining of strains TM108 (*ΔpilA*), EM747 (*ΔpilB*), RM216 (*ΔsgmX*) and EM589 (*ΔpilT*) on soft agar plate containing Congo Red.

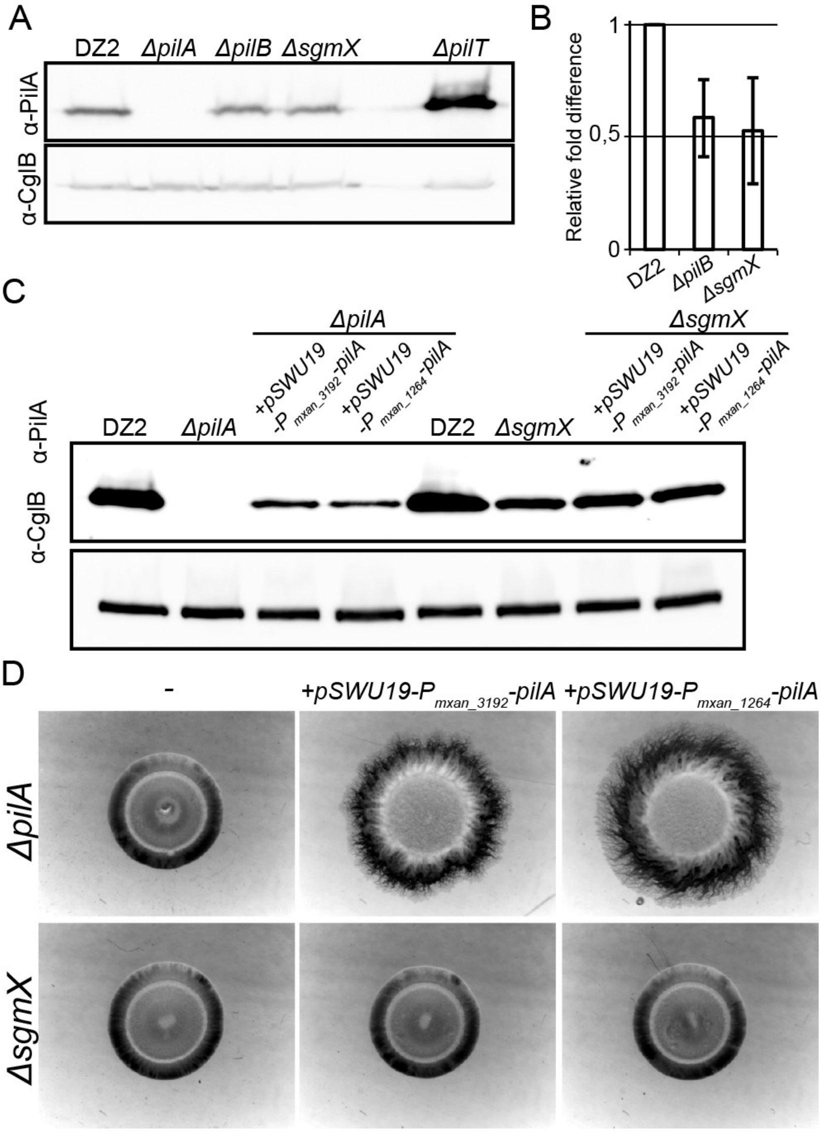

Fig. S5. Ectopic expression of PilA protein does not restore S-motility of a *sgmX* strain. (A) Western blot analysis with primary antibody directed against PilA (top) and CglB (bottom) proteins of cells from strains DZ2 (*Wild type*), TM108 (*ΔpilA*), EM747 (*ΔpilB*), RM216 (*ΔsgmX*) and EM589 (*ΔpilT*). (B) Relative fold difference of PilA protein concentration analyses by western in cells of strains DZ2 (*Wild type*), EM747 (*ΔpilB*), RM216 (*ΔsgmX*). The result represents the average of at least 2 independent experiments and associated standard deviation of the mean. (C) Western blot analysis with primary antibody directed against PilA (top) and CglB (bottom) proteins of cells from strains DZ2 (*Wild type*), TM108 (*ΔpilA*), RM403 (*ΔpilA pSWU19-P_{mxan_3192}-pilA*), RM404 (*ΔpilA pSWU19-P_{mxan_1254}-pilA*), RM216 (*ΔsgmX*), RM406 (*ΔsgmX pSWU19-P_{mxan_3192}-pilA*) and RM407 (*ΔsgmX pSWU19-P_{mxan_1254}-pilA*). D) Motility phenotypic assay of strains TM108 (*ΔpilA*; top left), RM403 (*ΔpilA pSWU19-P_{mxan_3192}-pilA*; top center), RM404 (*ΔpilA pSWU19-P_{mxan_1254}-pilA*; top right), RM216 (*ΔsgmX*; bottom left), RM406 (*ΔsgmX pSWU19-P_{mxan_3192}-pilA*; bottom center), RM407 (*ΔsgmX pSWU19-P_{mxan_1254}-pilA*; bottom right) on soft agar plate.

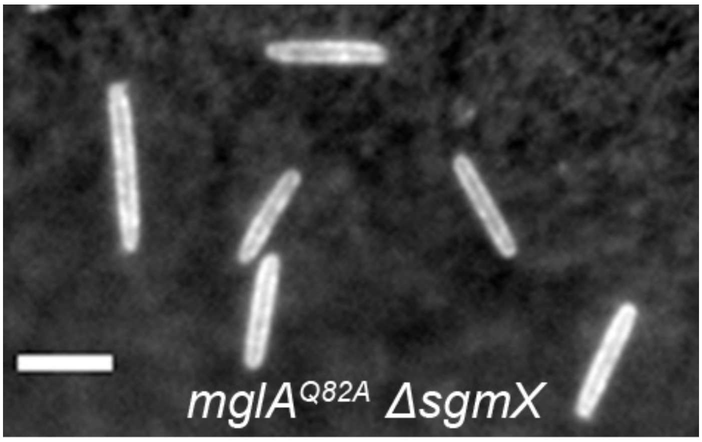

Fig. S6. *sgmX* mutation is epistastic to an *mglA*^{Q82A} variant on Tfpa machines activation. TIRF microscopy images of labelled Tfpa pilin of the strain RM402 (*mgla*^{Q82A} *ΔsgmX att*^{mx8}::P_{pilA}-*pilA*^{D71C}; e). Scale bar: 4 µm. See also SI Appendix, Movie S10.

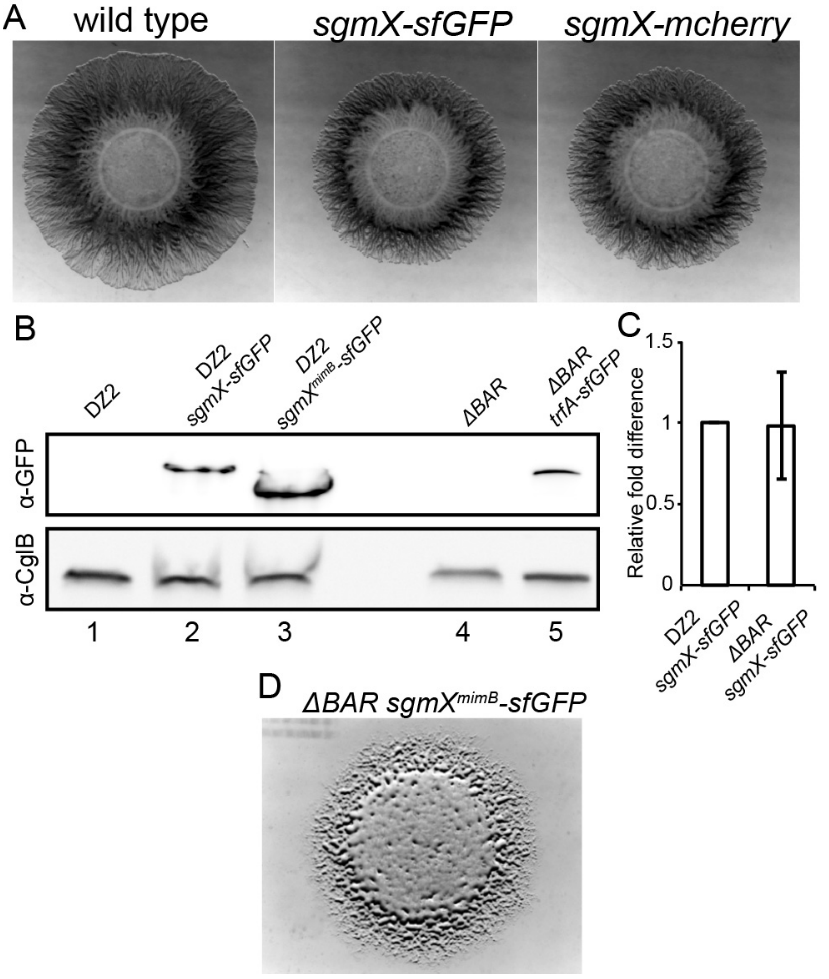

Fig. S7. Motility phenotypes and concentrations of fusion proteins SgmX-sfGFP, SgmX^{mimB}-sfGFP and SgmX-mcherry. (A) Motility phenotypic assay of strains DZ2 (*Wild type;* left)*,* RM190 (*sgmX-sfGFP;* center) and RM346 (*sgmX-mcherry;* right) on soft agar plate. (B) Western blots analysis with primary antibodies directed against GFP (top) and CglB (bottom) proteins of cells from strains DZ2 (*Wild type;* 1), RM190 (*sgmX-sfGFP;* 2), RM288 (*sgmX^{mimB}-sfGFP*; 3*)*, TM500 (*ΔBAR*; 4) and RM275 (*ΔBAR sgmX-sfGFP*; 5). (C) Relative fold difference of SgmX-sfGFP protein concentration analysed by western blot (B) in cells of strains RM190 (DZ2 *sgmX-sfGFP*) and RM275 (*ΔBAR sgmX-sfGFP*)*.* The result represents the average of 3 independent experiments and associated standard error of the mean. (D) Motility phenotypic assay of the strain RM260 (*ΔBAR sgmX^{mimB}-sfGFP)* on hard agar plate*.*

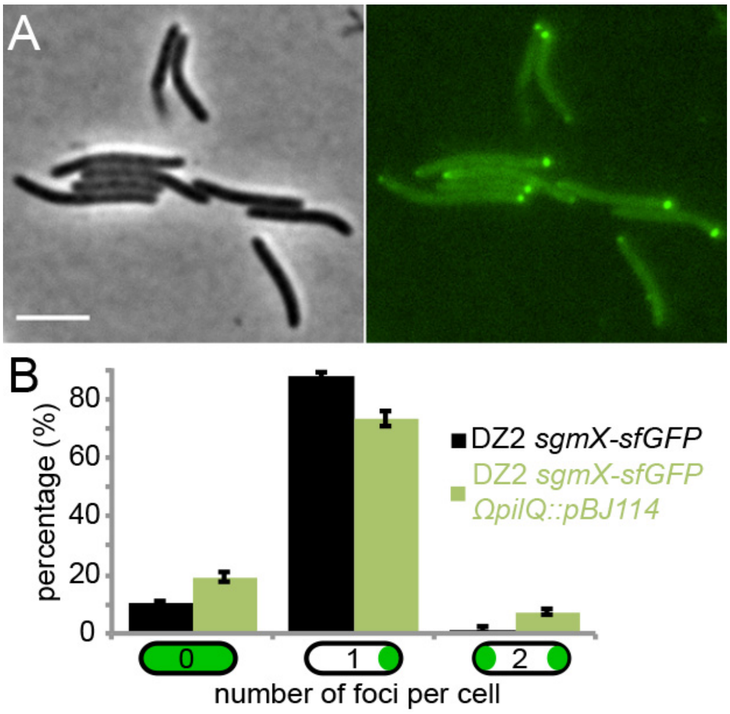

Fig. S8. Tfpa machine is not involved in SgmX polar localisation. (A) Phase contrast (left) and corresponding epifluorescence (right) images of the strain RM399 (*sgmX-sfGFP ΩpilQ::pBJ114*). Scale bar: 3 µm. (B) Histogram representing the proportion of cells with no (0), one (1) or two (2) SgmX-sfGFP foci per cell in strains RM190 (*sgmX-sfGFP;* black*,* n= 1384) and RM399 (*sgmX-sfGFP ΩpilQ::pBJ114;* green*,* n= 1087) The result represents the average proportion of n cells of 2 independent experiments and associated standard deviation of the mean.

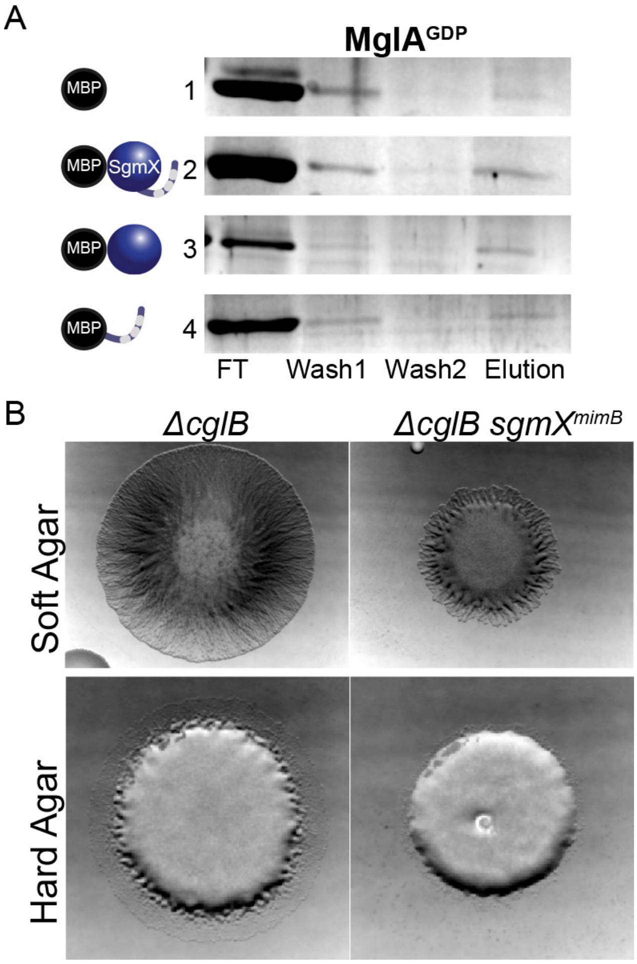

Fig. S9. Interaction between MglA-GTP and SgmX-C$_{Ter}$ is important S-motility. (A) Pull-down experiment of purified MglA-6His pre-incubated with GDP with purified MalE (1), MalE-SgmX$^{D2\text{-}L1060}$ (2), MalE-SgmX$^{D2\text{-}D853}$ (3) or MalE-SgmX$^{A813\text{-}L1060}$ (4) bound to amylose resin. The different lanes represent: Flow through (FT), the unbound MglA; Wash (W1, W2), washes with buffer; and Elution, MglA bound to SgmX. Samples were migrated on SDS-PolyAcrylamide Gel and protein bands were revealed by coomasie blue staining. (B) Motility phenotypic assay of strains TM913 (*ΔcglB;* left) *and* RM246 (*ΔcglB sgmX^{mimB};* right) on soft (top) and hard (bottom) agar plate.

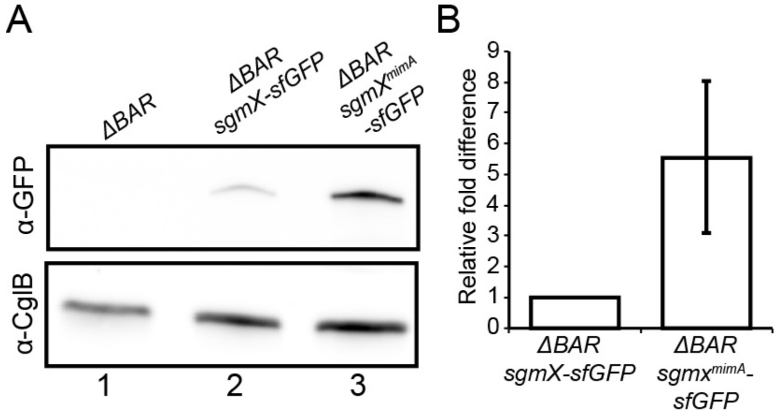

Fig. S10. MimA variant increases SgmX-sfGFP protein concentration. (A) Western blots analysis with primary antibodies directed against GFP (top) and CglB (bottom) proteins of cells from strains TM500 (*ΔBAR;* left), RM275 (*ΔBAR sgmX-sfGFP;* center) and RM192 (*ΔBAR sgmX^{mimA}-sfGFP;* right). (B) Relative fold difference of SgmX-sfGFP protein concentration analysed by western blot (A) in cells of strains RM275 (*ΔBAR sgmX-sfGFP*) and RM192 (*ΔBAR sgmX^{mimA}-sfGFP*). The result represents the average of 2 independent experiments and associated standard deviation of the mean.

**Table S1. Bacterial strains used in this study**		
Strain	Relevant Genotype (comments)	Reference
M. xanthus		
DZ2	Wild type	Laboratory collection
TM41	DZ2 *ΔmglA*	Laboratory collection
TM108	DZ2 *ΔpilA*	Laboratory collection
TM500	DZ2 *ΔromR ΔmglBA* (*ΔBAR*)	(7)
TM913	DZ2 *ΔcglB*	Laboratory collection
RM55	TM500 (*ΔBAR*) *mimA* (original *mimA* supressor strain)	This study
RM77	TM500 (*ΔBAR*) *mimB* (original *mimB* supressor strain)	This study
RM83	RM55 *pilA::tet*	This study
RM98	RM55 *cglB::Kn*	This study
RM182	DZ2 *ΔcglB ΔsgmX*	This study
RM185	DZ2 *Δmxan_5765*	This study
RM187	DZ2 *Ωmxan_5764::pBJ114*	This study
RM190	DZ2 *sgmX-sfGFP*	This study
RM192	RM55 *sgmX-sfGFP*	This study
RM194	DZ2 *ΔmglA sgmX-sfGFP*	This study
RM216	DZ2 *ΔsgmX*	This study
RM244	TM500 (*ΔBAR*) $mxan\_5766^{mimB}$ (Backcross of the *mimB* mutation into the strain TM500)	This study
RM246	DZ2 *ΔcglB* $sgmX^{mimB}$	This study
RM260	TM500 $sgmX^{mimB}$*-sfGFP*	This study
RM275	TM500 *sgmX-sfGFP*	This study
RM288	DZ2 $sgmX^{mimB}$*-sfGFP*	This study
RM310	TM500 (*ΔBAR*) $mxan\_5766^{mimA}$ (Backcross of the *mimA* mutation into the strain TM500)	This study
RM346	DZ2 *sgmX-mcherry*	This study
RM349	DZ2 *sgmX-mcherry mglA-YFP* att_{Mx8}*::pSWU30-*P_{mglA}*-mglA*	This study
RM353	DK1622 $mglA^{Q82A}$ *sgmX-sfGFP*	This study
RM375	RM55 *aglZ::Kn*	This study
RM382	DZ2 *ΔcglB ΔpilA* att_{Mx8}*::pSWU19-*P_{pilA}*-*$pilA^{D71C}$	This study
RM384	DZ2 att_{Mx8}*::pSWU19-*P_{pilA}*-*$pilA^{D71C}$	This study
RM386	DK1622 $mglA^{Q82A}$ att_{Mx8}*::pSWU19-*P_{pilA}*-*$pilA^{D71C}$	This study
RM388	TM500 att_{Mx8}*::pSWU19-*P_{pilA}*-*$pilA^{D71C}$	This study
RM390	DZ2 *ΔmglA* att_{Mx8}*::pSWU19-*P_{pilA}*-*$pilA^{D71C}$	This study
RM391	DZ2 *ΔsgmX* att_{Mx8}*::pSWU19-*P_{pilA}*-*$pilA^{D71C}$	This study
RM392	DZ2 *ΔpilB* att_{Mx8}*::pSWU19-*P_{pilA}*-*$pilA^{D71C}$	This study
RM393	DZ2 $sgmX^{mimB}$*-mcherry* att_{Mx8}*::pSWU19-*P_{pilA}*-*$pilA^{D71C}$	This study
RM394	RM310 att_{Mx8}*::pSWU19-*P_{pilA}*-*$pilA^{D71C}$	This study
RM395	RM244 att_{Mx8}*::pSWU19-*P_{pilA}*-*$pilA^{D71C}$	This study
RM399	DZ2 *sgmX-sfGFP ΩpilQ::pBJ114*	This study
RM402	DK1622 $mglA^{Q82A}$ *ΔsgmX* att_{Mx8}*::pSWU19-*P_{pilA}*-*$pilA^{D71C}$	This study
RM403	DZ2 *ΔpilA* att_{Mx8}*::pSWU19-*P_{mxan_3192}*-pilA*	This study

RM404	DZ2 *ΔpilA* att_{Mx8}::*pSWU19*-P_{mxan_1264}-*pilA*	This study
RM406	DZ2 *ΔsgmX* att_{Mx8}::*pSWU19*-P_{mxan_3192}-*pilA*	This study
RM407	DZ2 *ΔsgmX* att_{Mx8}::*pSWU19*-P_{mxan_1264}-*pilA*	This study
EM589	DZ2 *ΔpilT*	Laboratory collection
EM617	DZ2 *ΔdifD ΔdifG*	Laboratory collection
EM746	DZ2 *ΔdifD ΔdifG ΔsgmX*	This study
EM747	DZ2 *ΔpilB*	Laboratory collection
E. coli		
BL21 pLysS	F–, *omp*T, *hsd*S_B (r_B–, m_B–), *dcm*, *gal*, λ(DE3), pLysS, Cm^r	Laboratory collection

Table S2. Plasmids used in this study

Plasmid	Relevant Genotype	Reference
pBJ114	Used to create deletions or insertions, *galK*, Kan	Laboratory collection
pBJ114-*mxan_5766*^{mimA}	pBJ114 with insertion cassette for *mxan_5766*^{mimA}	This study
pBJ114-*ΩcglB*	pBJ114 with insertion cassette for *cglB*	(8)
pBJ114-Δ*sgmX*	pBJ114 with deletion cassette for *sgmX*	This study
pBJ114-Δ*mxan_5765*	pBJ114 with deletion cassette for *mxan_5765*	This study
pBJ114-*Ωmxan_5764*	pBJ114 with insertion cassette for *mxan_5764*	This study
pBJ114-*sgmX-sfGFP*	pBJ114 with insertion cassette for the creation of *sgmX-sfGFP*	This study
pBJ114-*sgmX-mcherry*	pBJ114 with insertion cassette for the creation of *sgmX-mcherry*	This study
pBJ114-*ΩpilQ*	pBJ114 with insertion cassette for *pilQ*	This study
pBJ114-*sgmX*^{mimB}-*sfGFP*	pBJ114 with insertion cassette for the creation of *sgmX*^{mimB}-*sfGFP*	This study
pBJ114-*sgmX*^{mimB}-*mcherry*	pBJ114 with insertion cassette for the creation of *sgmX*^{mimB}-*mcherry*	This study
pSWU19	Used for insertions at Mx8 phage *attB* site, Kan	Laboratory collection
pSWU19-P_{pilA}-*pilA*^{D71C}	pSWU19 to express P_{pilA}-*pilA*^{D71C} variant at Mx8 phage *attB* site	This study
pSWU19-P_{mxan_1264}-*pilA*	pSWU19 to express P_{mxan_1264}-*pilA* at Mx8 phage *attB* site	This study
pSWU19-P_{mxan_3102}-*pilA*	pSWU19 to express P_{mxan_3192}-*pilA* at Mx8 phage *attB* site	This study
pSWU30-P_{mglA}-*mglA*	pSWU30 to express P_{mglA}-*mglA* at Mx8 phage *attB* site, Tet	(7)
pMal-C2G	Used to purifie MalE fused protein, bla	Laboratory collection

pMal-C2G-*sgmX*	Used to purifie MalE-SgmX protein	This study
pMal-C2G-*sgmX*$^{\Delta MBD}$	Used to purifie MalE-SgmX$^{D2\text{-}D853}$ protein	This study
pMal-C2G-*sgmX*^{MBD}	Used to purifie MalE-SgmX$^{A813\text{-}L1060}$ protein	This study
pET28a-*mglA*$_{\text{-His6}}$	Used to purifie MglA-His6 protein	(7)

Kan, Kanamycin bla, β-lactamase

Movie S1. Movie_S1.avi

Time-lapse series showing cell motility and labelled Tfpa pilin filaments of the strain RM384 (DZ2 *att*^{mx8}::*P*$_{pilA}$-*pilA*^{D71C}) observed by TIRF microscopy, from which the panels in Fig. 1A were obtained. Fluorescent images were acquired automatically every 2 s for 72 s. Scale bar: 2 µm.

Movie S2. Movie_S2.avi

Time-lapse series showing cell pole inversion of labelled Tfpa pilin filaments of the strain RM384 (DZ2 *att*^{mx8}::*P*$_{pilA}$-*pilA*^{D71C}) observed by TIRF microscopy. Fluorescent images were acquired automatically every 2 s for 2 min. Scale bar: 2 µm.

Movie S3. Movie_S3.avi

Time-lapse series showing cell pole inversion of polar cluster enrichment of labelled Tfpa pilin of the strain RM384 (DZ2 *att*^{mx8}::*P*$_{pilA}$-*pilA*^{D71C}) observed by TIRF microscopy, from which the panels in Fig. 1B were obtained. Fluorescent images were acquired automatically every 2 s for 3 min. Scale bar: 2 µm.

Movie S4. Movie_S4.avi

Time-lapse series showing a cell with labelled Tfpa pilin filaments of the strain RM390 (*ΔmglA att*^{mx8}::*P*$_{pilA}$-*pilA*^{D71C}) observed by TIRF microscopy. Fluorescent images were acquired automatically every 2 s for 2 min. Scale bar: 2 µm.

Movie S5. Movie_S5.avi

Time-lapse series showing cells with labelled Tfpa pilin filaments of the strain RM386 (*mglA*^{Q82A} *att*^{mx8}::*P*$_{pilA}$-*pilA*^{D71C}) observed by TIRF microscopy, from which the panel in Figure 2f were obtained. Fluorescent images were acquired automatically every 2 s for 2 min. Scale bar: 4 µm.

Movie S6. Movie_S6.avi

Time-lapse series showing cell motility of the strain RM55 (*ΔBAR*^{mimA}), from which the panels in SI Appendix Fig. S2C were obtained. Phase-contrast images were acquired automatically every 1 min for 3 hr. Scale bar: 3 µm.

Movie S7. Movie_S7.avi

Time-lapse series showing cell motility of the strain TM500 (*ΔBAR),* from which the panels in SI Appendix Fig. S2D were obtained. Phase-contrast images were acquired automatically every 1 min for 3 hr. Scale bar: 3 µm.

Movie S8. Movie_S8.avi

Time-lapse series showing a cell with labelled Tfpa pilin filaments of the strain RM391 (*ΔsgmX* $att^{mx8}::P_{pilA}\text{-}pilA^{D71C}$) observed by TIRF microscopy. Fluorescent images were acquired automatically every 2 s for 2 min. Scale bar: 2 µm.

Movie S9. Movie_S9.avi

Time-lapse series showing a cell with labelled Tfpa pilin filaments of the strain RM392 (*ΔpilB* $att^{mx8}::P_{pilA}\text{-}pilA^{D71C}$) observed by TIRF microscopy. Fluorescent images were acquired automatically every 2 s for 2 min. Scale bar: 2 µm.

Movie S10. Movie_S10.avi

Time-lapse series showing a cell with labelled Tfpa pilin filaments of the strain RM402 ($mglA^{Q82A}$ *ΔsgmX* $att^{mx8}::P_{pilA}\text{-}pilA^{D71C}$) observed by TIRF microscopy. Fluorescent images were acquired automatically every 2 s for 2 min. Scale bar: 3 µm.

Movie S11. Movie_S11.avi

Time-lapse series showing a pole-to-pole dynamics of SgmX-sfGFP in a single reversing cell of the strain RM190 (*sgmX-sfGFP*), from which the panels in Figure 3C were obtained. Phase-contrast and fluorescent images were acquired automatically every 10 s for 140 s. Scale bar: 2 µm.

Movie S12. Movie_S12.avi

Time-lapse series showing $SgmX^{mimB}$-mcherry polar localisation (bottom) in a cell with labelled Tfpa pilin filaments (top) of the strain RM393 ($sgmX^{mimB}$*-mcherry* $att^{mx8}::P_{pilA}\text{-}pilA^{D71C}$) observed by TIRF microscopy, from which the panels in Fig. 4F were obtained. Fluorescent images were acquired automatically every 2 s for 2 min. Scale bar: 3 µm.

Movie S13. Movie_S13.avi

Time-lapse series showing the correlation between the uni or bi-polar localisation of $SgmX^{mimB}$-mcherry (bottom) and the presence of polar pilin cluster (top) of cells with labelled Tfpa pilin filaments of the strain RM393 ($sgmX^{mimB}$*-mcherry* $att^{mx8}::P_{pilA}\text{-}pilA^{D71C}$) observed by TIRF microscopy. Fluorescent images were acquired automatically every 2 s for 2 min. Scale bar: 3 µm.

Movie S14. Movie_S14.avi

Time-lapse series showing a pole-to-pole dynamics of $SgmX^{mimB}$-sfGFP in a single reversing cell of the strain RM288 (*$sgmX^{mimB}$-sfGFP*), from which the panels in Fig. 4E were obtained. Fluorescent images were acquired automatically every 30 s for 20 min. Scale bar: 2 µm.

References

1. J. Sambrook, Fritsch, E.F., and Maniatis, T, Molecular cloning: A Laboratory Manual. *New YorK: Cold Spring Harbor: Cold Spring Harbor Laboratory Press* (1989).
2. V. H. Bustamante, I. Martinez-Flores, H. C. Vlamakis, D. R. Zusman, Analysis of the Frz signal transduction system of *Myxococcus xanthus* shows the importance of the conserved C-terminal region of the cytoplasmic chemoreceptor FrzCD in sensing signals. *Mol Microbiol* **53**, 1501-1513 (2004).
3. J. Munoz-Dorado *et al.*, Transcriptome dynamics of the Myxococcus xanthus multicellular developmental program. *eLife* **8** (2019).
4. M. Guzzo *et al.*, Evolution and Design Governing Signal Precision and Amplification in a Bacterial Chemosensory Pathway. *PLoS Genet* **11**, e1005460 (2015).
5. A. Ducret, E. M. Quardokus, Y. V. Brun, MicrobeJ, a tool for high throughput bacterial cell detection and quantitative analysis. *Nature Microbiol* **1**, 16077 (2016).
6. L. Zimmermann *et al.*, A Completely Reimplemented MPI Bioinformatics Toolkit with a New HHpred Server at its Core. *Journal of molecular biology* **430**, 2237-2243 (2018).
7. Y. Zhang, M. Franco, A. Ducret, T. Mignot, A bacterial Ras-like small GTP-binding protein and its cognate GAP establish a dynamic spatial polarity axis to control directed motility. *PLoS biology* **8**, e1000430 (2010).
8. A. Ducret, M. P. Valignat, F. Mouhamar, T. Mignot, O. Theodoly, Wet-surface-enhanced ellipsometric contrast microscopy identifies slime as a major adhesion factor during bacterial surface motility. *Proc Natl Acad Sci U S A* **109**, 10036-10041 (2012).
